# Liquid biopsy, multi-cancer early detection, and artificial intelligence: new frontiers in cancer screening from a technological and immunological perspective

**DOI:** 10.3389/fimmu.2026.1854718

**Published:** 2026-06-16

**Authors:** Dong Wu, Qing Yu, Qixiang Wu, Xiaoying Wang, Haojie Wei, Fengli Li, Guangli Li, Xiaowu Wang, Xiaojuan Wang

**Affiliations:** 1 Department of Clinical Pharmacy, Fuyang People’s Hospital, Fuyang, China; 2 Department of Pharmacy, Tongling People’s Hospital, Tongling Academy of Medical Sciences, Tongling, China; 3 Inflammation and Immune Mediated Diseases Laboratory of Anhui Province; School of Pharmaceutical Sciences, Anhui Medical University, Hefei, China; 4 Department of Clinical Pharmacy, Fuyang Cancer Hospital, Fuyang, China; 5 Department of Clinical Laboratory, The Second People’s Hospital of Fuyang City, Fuyang Infectious Disease Clinical College of Anhui Medical University, Fuyang, China

**Keywords:** artificial intelligence, clinical validation, early cancer detection, liquid biopsy, multi-cancer early detection, tumor immune interactions

## Abstract

Early cancer detection remains a central challenge in oncology because many lethal tumors are diagnosed after curative opportunities have narrowed, whereas current organ-specific screening methods cover only a limited number of cancer types and may be constrained by invasiveness, cost, accessibility or stage-dependent sensitivity. Liquid biopsy, multi-cancer early detection (MCED) and artificial intelligence (AI) are rapidly reshaping this field, but their clinical implications require careful interpretation. This review critically evaluates major liquid-biopsy analytes, including circulating tumor DNA, cell-free DNA methylation and fragmentomics, circulating tumor cells, extracellular vesicles, non-coding RNAs, tumor-educated platelets and multi-omics signatures, with emphasis on intended use, clinical maturity, tissue-of-origin value and translational limitations. A distinctive feature of this review is the integration of tumor-derived signals with host-response and immunological readouts, including peripheral blood mononuclear cell-based monitoring, immune-cell-derived extracellular vesicles, exosomal immune-checkpoint molecules and inflammatory confounders, thereby framing liquid biopsy as both a cancer-detection tool and a window into tumor–immune interactions. We further discuss MCED as a clinical care pathway rather than an isolated blood test, highlighting the importance of positive and negative predictive values, cancer prevalence, diagnostic-resolution pathways, false-positive workup, overdiagnosis, mortality benefit, cost-effectiveness and equitable access. The role of AI is examined in relation to model development, multimodal fusion, tissue-of-origin prediction, calibration, interpretability, bias, generalizability and clinical implementation. Across these technologies, a key translational message is that technical detectability is not equivalent to clinical readiness. While selected assays have entered defined clinical or guideline-supported settings, many emerging biomarkers and AI-enabled models remain investigational or translational. Future progress will depend on standardized workflows, prospective validation in representative populations, evidence of clinical utility, regulatory and ethical oversight, and integration with established screening and diagnostic systems.

## 1 Introduction

Cancer remains a leading cause of premature death worldwide and a major public health and socioeconomic challenge (1, 2). The International Agency for Research on Cancer estimated nearly 20 million new cancer cases and 9.7 million cancer deaths globally in 2022, and projections suggest that annual new cases may exceed 35 million by 2050 (1–4). Prognosis is strongly stage-dependent; for example, population-based data for colorectal cancer (CRC) show that 5-year relative survival approaches 90% for stage I disease but falls to around 15% for stage IV disease (5). These observations underscore the importance of detecting cancer at an earlier, more treatable stage.

Early cancer screening can reduce cancer-related mortality by identifying malignancies before clinical progression (6). However, existing screening and diagnostic strategies have important limitations. Imaging methods, including magnetic resonance imaging, computed tomography and ultrasound, often rely on structural or phenotypic changes that may appear relatively late in tumor evolution, whereas tissue biopsy is invasive and unsuitable for broad population-level screening (7). Conventional serum biomarkers, including prostate-specific antigen (PSA), carbohydrate antigen 19-9 (CA19-9) and cancer antigen 125 (CA-125), are clinically useful in selected contexts but often lack sufficient specificity for early cancer detection (8–10). Their levels may be influenced by benign prostatic, inflammatory, hepatobiliary or gynecological conditions, leading to false-positive results, unnecessary diagnostic procedures and overdiagnosis (11–13).

Recent advances in liquid biopsy, multi-cancer early detection (MCED) tests and artificial intelligence (AI) are reshaping cancer screening. Liquid biopsy analyzes biomarkers such as cell-free DNA (cfDNA), circulating tumor DNA (ctDNA), extracellular vesicles and other molecular or cellular signals in blood and other biofluids, providing a minimally invasive complement to tissue biopsy for molecular detection and longitudinal assessment in selected clinical contexts (14–16). MCED platforms aim to detect signals from multiple cancer types through a single blood test and may extend detection to cancers that currently lack established screening programs, but their sensitivity remains stage- and tumor-type-dependent, particularly for stage I disease, and their effects on late-stage cancer incidence, mortality and cost-effectiveness remain to be confirmed (17, 18). AI-based methods, especially machine learning (ML) and deep learning (DL), may support image interpretation, biomarker discovery and multimodal data integration, but their clinical value depends on external validation, calibration, interpretability and integration into real-world workflows (19–21).

**Figure 1:**
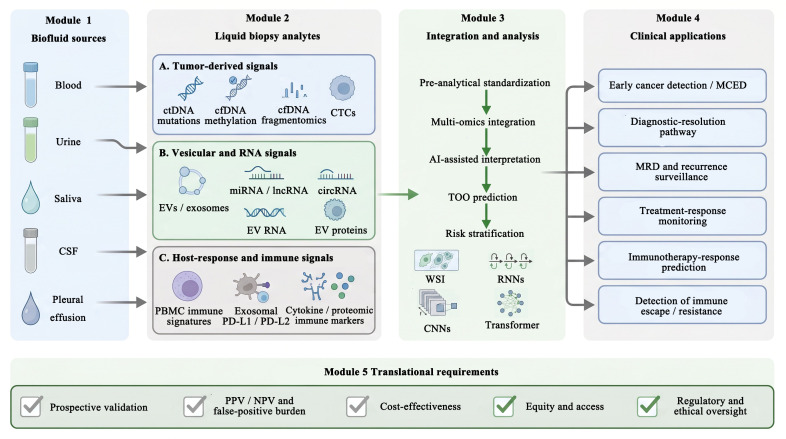
Integrated liquid-biopsy framework linking biofluid sources, analyte classes, AI-assisted interpretation, clinical applications and translational requirements in cancer screening and monitoring. The schematic illustrates a five-module framework. Module 1 lists representative biofluid sources, including blood, urine, saliva, CSF and pleural effusion. Module 2 summarizes major liquid-biopsy analyte classes, including tumor-derived signals, vesicular and RNA signals, and host-response or immune signals. Tumor-derived signals include ctDNA mutations, cfDNA methylation, cfDNA fragmentomics and circulating tumor cells. Vesicular and RNA signals include EVs / exosomes, miRNAs, lncRNAs, circRNAs, EV RNA and EV proteins. Host-response and immune signals include PBMC immune signatures, exosomal PD-L1 / PD-L2 and cytokine/proteomic immune markers. Module 3 highlights the analytical and interpretive layer required for clinical translation, including pre-analytical standardization, multi-omics integration, AI-assisted interpretation, TOO prediction and risk stratification. Module 4 presents representative clinical applications, including early cancer detection and MCED, diagnostic-resolution pathways, MRD and recurrence surveillance, treatment-response monitoring, immunotherapy-response prediction and detection of immune escape or resistance. Module 5 summarizes key translational requirements for responsible implementation, including prospective validation, assessment of PPV / NPV and false-positive burden, cost-effectiveness, equity and access, and regulatory and ethical oversight. AI, artificial intelligence; cfDNA, cell-free DNA; circRNA, circular RNA; CTCs, circulating tumor cells; ctDNA, circulating tumor DNA; EV, extracellular vesicle; lncRNA, long non-coding RNA; MCED, multi-cancer early detection; miRNA, microRNA; MRD, minimal residual disease; NPV, negative predictive value; PBMC, peripheral blood mononuclear cell; PD-L1 / PD-L2, programmed death-ligand 1/2; PPV, positive predictive value; TOO, tissue-of-origin; CSF, cerebrospinal fluid.

This review differs from previous broad summaries in three ways. First, it evaluates liquid-biopsy analytes through a clinically oriented framework that distinguishes intended use, clinical maturity, early-stage sensitivity, tissue-of-origin value and major translational limitations. Second, it integrates tumor-derived signals with host-response and immunological readouts, including peripheral blood mononuclear cell (PBMC)-based immune signatures, immune-cell-derived extracellular vesicles (EVs), tumor-educated platelets and exosomal immune-checkpoint molecules. This framing allows liquid biopsy to be interpreted not only as a cancer-detection technology, but also as a window into tumor–immune interactions, inflammatory confounding, immune escape and immunotherapy-response monitoring. Third, it critically examines MCED and AI as implementation challenges that require external validation, diagnostic-resolution pathways, cost-effectiveness, equity assessment, interpretability and regulatory oversight. By combining technological, immunological and translational perspectives, this review provides a focused framework for understanding how liquid biopsy, MCED and AI may be responsibly integrated into future cancer-screening strategies. The conceptual framework of this review, integrating biofluid sources, major liquid-biopsy analyte classes, AI-assisted interpretation, clinical applications and translational requirements, is summarized in **Figure 1**.

## 2 Liquid biopsy as a clinically stratified framework

### 2.1 Intended use, diagnostic performance and clinical maturity

Liquid biopsy refers to the analysis of tumor-derived, tumor-associated and host-response biomarkers in blood, urine, saliva, cerebrospinal fluid, pleural effusions and other accessible biofluids. These biomarkers include circulating tumor cells (CTCs), ctDNA, cfDNA, microRNAs (miRNAs), EVs, other cell-free nucleic acids and circulating immune-cell readouts such as PBMC-based immune signatures (22–25). This broader “beyond blood” concept, emphasized by Bao et al., is important because different tumor types and anatomical sites may release more informative signals into different biofluid compartments, thereby expanding the clinical scope of liquid biopsy across early detection, diagnostic workup, treatment monitoring, recurrence surveillance and personalized therapeutic decision-making (24).

Liquid-biopsy applications should be interpreted according to intended clinical use. Screening or early detection refers to testing asymptomatic or high-risk individuals before clinical diagnosis and requires very high specificity, low false-positive burden, validated diagnostic workup pathways and evidence of stage shift or outcome benefit. Diagnostic use, minimal residual disease (MRD) monitoring, recurrence surveillance, treatment-response monitoring and prognostic risk stratification require different validation endpoints and clinical decision pathways. Therefore, diagnostic performance reported in advanced-disease monitoring should not be directly extrapolated to population-level early cancer screening.

When reporting diagnostic performance, sensitivity, specificity, area under the receiver operating characteristic curve (AUC) and related metrics should be interpreted in the context of study design, cohort type, sample size, cancer prevalence, stage distribution, validation status and intended use. Results from retrospective case-control studies, disease-enriched cohorts or comparisons with healthy controls may overestimate performance compared with real-world screening populations, where cancer prevalence is lower, benign mimics are more common and false-positive findings have greater downstream consequences. Accordingly, high diagnostic accuracy in discovery or internal-validation cohorts should be regarded as hypothesis-generating unless supported by independent external validation and, for screening applications, prospective evaluation in asymptomatic or clinically relevant high-risk populations.

The clinical maturity of liquid-biopsy analytes also differs substantially. Some approaches have entered defined clinical or guideline-supported settings, including CTC enumeration for prognostic assessment in selected metastatic cancers (26, 27), methylated SEPT9 testing for colorectal cancer screening in defined populations (28), and urine exosomal RNA testing for prostate biopsy risk stratification (29). By contrast, cfDNA fragmentomics, tumor-educated platelets (TEPs), circular RNAs (circRNAs), many EV cargo markers and multi-omics signatures remain largely translational or exploratory and require standardized workflows, external validation and evidence of clinical utility before routine screening use (30–32). The biological characteristics of major liquid-biopsy analytes are summarized in **Table 1**, and their clinical maturity and translational interpretation are summarized in **Table 2**.

**Table 1:** Characteristics of major liquid-biopsy analytes for early cancer detection.

Analyte class	Main source/biofluids	Signal level	Main advantages	Main limitations	Clinical maturity/early-detection relevance	Ref
CTCs	Blood, cerebrospinal fluid, urine and other biofluids	Intact cellular analyte	Provides viable or intact tumor cells for phenotypic, genomic, transcriptomic and functional characterization; may reflect invasion, metastatic potential and tumor heterogeneity	Extremely rare in early-stage disease; enrichment bias; epithelial-marker dependence; isolation and reporting thresholds are not fully standardized	Established mainly for prognostic assessment in selected metastatic cancers; early-detection use remains translational	(26, 27, 34, 35)
cfDNA / ctDNA	Blood, cerebrospinal fluid, saliva, urine and other biofluids	Cell-free nucleic-acid fragments	Supports mutation, methylation, copy-number and fragmentomic analysis; compatible with repeated sampling and tissue-of-origin inference	Low tumor fraction in early-stage disease; clonal hematopoiesis and non-tumor cfDNA background may cause false-positive or ambiguous signals	Clinically mature for molecular profiling and monitoring in established cancer; selected cfDNA-based tests have entered defined screening settings, but standalone early screening remains context-dependent	(33, 37–39)
EVs / exosomes	Blood, urine, saliva, cerebrospinal fluid, pleural effusions and other biofluids	Vesicular analyte carrying proteins, lipids, DNA and RNA	Stable cargo protected by lipid bilayer; reflects tumor, stromal and immune-cell communication; suitable for multi-cargo and multi-omics analysis	Isolation purity, normalization, vesicle subtyping and reporting remain insufficiently standardized; sensitivity and specificity depend strongly on cargo and platform	Selected urine exosomal RNA assays have entered clinical decision-support settings; most EV-based early-detection biomarkers remain exploratory or translational	(29, 32)
TEPs	Peripheral blood platelets	Host-response cellular analyte	Platelet RNA and splicing profiles may reflect tumor–platelet interactions and support cancer detection or tissue-of-origin inference	Susceptible to platelet activation, inflammation, thrombosis and handling artifacts; requires careful RNA-seq normalization and validation	Promising but still exploratory/translational for early detection; not established for routine screening	(40–42)
Circulating non-coding RNAs	Blood, urine, saliva, cerebrospinal fluid and EV-enriched fractions	miRNA, lncRNA and circRNA signals	Stable and measurable in biofluids; may reflect tumor biology, tissue context and tumor–host interactions; useful in multi-marker panels	Single RNA markers often lack cancer specificity and may be influenced by hemolysis, inflammation, tissue injury and sample handling	Mostly exploratory; diagnostic value is likely greatest in validated multi-marker or multi-omics panels rather than standalone tests	(43–46)
PBMC-based immune signatures	Peripheral blood mononuclear cells	Host immune-cell readout	Provides systemic immune information and may capture treatment-associated immune activation or tumor–immune interactions	Cell-subset heterogeneity, isolation variability and therapy-related immune changes complicate interpretation	Relevant mainly for immune monitoring and treatment-response assessment; early-detection role remains exploratory	(25, 36)
Integrated multi-omics signatures	Blood and other biofluids with combined molecular layers	Composite genomic, epigenomic, transcriptomic, proteomic, fragmentomic and clinical signals	Combines complementary analytes and may improve sensitivity, tissue-of-origin prediction and risk stratification	Missing data, batch effects, feature scaling, platform-specific noise and overfitting; requires external validation	Promising for MCED and risk stratification, but most models remain in retrospective validation or early prospective phases	(47, 48)

cfDNA, cell-free DNA; circRNA, circular RNA; CTCs, circulating tumor cells; ctDNA, circulating tumor DNA; DNA, deoxyribonucleic acid; EVs, extracellular vesicles; lncRNA, long non-coding RNA; MCED, multi-cancer early detection; miRNA, microRNA; PBMC, peripheral blood mononuclear cell; RNA, ribonucleic acid; TEPs, tumor-educated platelets.

**Table 2:** Clinical maturity, regulatory status and translational interpretation of major liquid-biopsy analytes.

Analyte/biomarker class	Regulatory/guideline status or evidence tier	More established or intended clinical use	Early cancer screening status	TOO value	Major translational limitations	Representative references
CTCs / CellSearch® system	FDA-cleared for CTC enumeration as an aid in monitoring patients with metastatic breast, colorectal and prostate cancers; not approved for population-level early cancer screening	Prognostic assessment and disease monitoring in selected metastatic cancers	Not established for population-level early screening	Moderate; intact cells allow phenotypic, genomic and functional analysis, but enrichment may bias captured cells	Rare in early-stage disease; EpCAM/enrichment bias; epithelial–mesenchymal transition may reduce capture; blood-volume dependence; lack of standardized early-screening thresholds	(26, 27)
SEPT9 methylation / Epi proColon	FDA-approved blood-based CRC screening test for defined adults ≥50 years at average CRC risk who have not completed recommended screening methods; positive results require diagnostic colonoscopy	Blood-based CRC screening option in defined settings, especially when standard screening is declined or not completed	Clinically implemented for CRC only, but not a replacement for colonoscopy/FIT and not an MCED test	Low; single-cancer CRC-oriented test, not broad tissue-of-origin classifier	Modest sensitivity/specificity compared with established CRC screening; no proven mortality-benefit evidence as a replacement strategy; repeat-use performance and integration issues	(28)
cfDNA-based CRC screening / Shield	FDA-approved blood-based CRC screening test for adults aged ≥45 years at average CRC risk who need CRC screening; single-cancer CRC screening test, not MCED; positive results require diagnostic colonoscopy	CRC screening in eligible average-risk adults aged ≥45 years; abnormal results require diagnostic colonoscopy	Clinically implemented for CRC only; not applicable to MCED	Low; designed for CRC detection rather than tissue-of-origin prediction	Lower sensitivity for advanced precancerous lesions than for CRC; positive results still require colonoscopy; long-term mortality benefit and optimal screening interval require continued evaluation	(39)
ctDNA mutations	Clinically implemented in oncology for molecular profiling, treatment selection, MRD assessment, recurrence surveillance and resistance monitoring in known cancers; not approved as standalone population screening	Tumor genotyping, targeted therapy selection, MRD and resistance monitoring in established cancer	Limited as standalone early-screening tool because tumor fraction is often extremely low in stage I disease	Low to moderate; many driver mutations are shared across tumor types and may not localize origin	Low tumor fraction, sequencing error, clonal hematopoiesis, need for matched leukocyte controls, unknown tumor genotype in asymptomatic screening	(49–51)
cfDNA methylation MCED / Galleri-like assays	Clinically validated / investigational-translational; Galleri has undergone large clinical validation and implementation studies, and GRAIL submitted a PMA application to the FDA in 2026, which was reported by GRAIL as accepted for FDA review; however, Galleri is not FDA-cleared or FDA-approved as a routine MCED screening test	Cancer-signal detection and tissue-of-origin prediction in validation and implementation studies	Promising but not established as a population-level screening standard.	High; methylation patterns are tissue- and cell-type informative	Stage-dependent sensitivity, especially limited for stage I disease; need evidence for clinical utility, mortality benefit, cost-effectiveness, diagnostic-resolution pathways and equitable implementation	(18, 52, 53)
cfDNA fragmentomics / DELFI-like approaches	Investigational / translational; not an FDA-approved screening test	Complementary cancer-signal detection and localization in research or validation studies	Promising but investigational for asymptomatic population screening	Moderate to high; fragment size, nucleosome positioning and chromatin-related patterns may support tissue inference	Sensitive to pre-analytical variation, inflammation, non-tumor cell death and computational model instability; requires prospective validation in asymptomatic screening populations	(30, 54)
ExoDx Prostate IntelliScore / urine exosomal RNA	Guideline-supported clinical decision-support assay for prostate biopsy risk stratification; not a general cancer-screening test	Risk stratification for high-grade prostate cancer and biopsy decision support	Not a population-level cancer screening test; prostate-specific decision-support use	Low to moderate; prostate-focused RNA signature	Intended for a defined prostate biopsy decision context; not MCED; performance depends on target population and PSA/clinical context	(29)
Other exosomes / extracellular-vesicle cargo markers	Mostly investigational / exploratory; selected assays have clinical decision-support use, but most EV cancer-detection biomarkers are not clinically established	Research-stage cancer detection, molecular profiling and tumor–immune interaction assessment	Not broadly established for routine screening	Moderate; depends on tumor specificity of cargo, vesicle source and biofluid compartment	Isolation variability, purity/yield trade-offs, normalization issues, pre-analytical variation, lack of standardized reporting; need MISEV-compliant validation	(29, 32)
miRNA / lncRNA / circRNA	Exploratory to translational; many candidate panels but few clinically established screening tests	Candidate diagnostic/prognostic panels; selected prostate RNA assays have decision-support relevance	Not established for population-level early screening	Low to moderate; some RNAs are tissue-enriched, but specificity is often limited	Hemolysis, inflammation, tissue injury, sample-processing effects, normalization challenges, limited prospective and external validation	(55–59)
Tumor-educated platelets / TEPs	Exploratory / translational; no established routine screening indication	Research-stage tumor detection, tumor localization and host-response assessment	Not established for routine screening	Moderate; platelet RNA profiles may support tumor-type classification	Platelet activation during handling, inflammatory/thrombotic confounding, low-input sequencing requirements, limited prospective validation	(31, 41, 42)
Multi-omics liquid biopsy	Proof-of-concept to early clinical validation; not established as a routine population screening test	Integrated cancer-signal detection, risk modeling and tissue-origin prediction in research/validation studies	Promising but not established as a population-level standard	Potentially high when combining methylation, fragmentomics, mutations, proteins, EV cargo and clinical variables	Analytical complexity, missing data, batch effects, platform-specific noise, feature scaling, high cost, unclear diagnostic workup burden, need for clinical utility and cost-effectiveness evidence	(60–63)
Metabolomics / microbiome-based markers	Exploratory	Research-stage adjunctive biomarkers	Not established for population-level screening	Usually low to moderate; signals may reflect organ-specific or systemic biology but are highly confounded	High biological variability, diet/drug/inflammation confounding, limited reproducibility and limited multicenter validation	(64, 65)

TOO, Tissue-of-origin; CCGA, Circulating Cell-free Genome Atlas; cfDNA, cell-free DNA; circRNA, circular RNA; CRC, colorectal cancer; CTCs, circulating tumor cells; ctDNA, circulating tumor DNA; DELFI, DNA evaluation of fragments for early interception; EpCAM, epithelial cell adhesion molecule; EV, extracellular vesicle; FDA, Food and Drug Administration; FIT, fecal immunochemical test; HCC, hepatocellular carcinoma; lncRNA, long non-coding RNA; MCED, multi-cancer early detection; miRNA, microRNA; MISEV, Minimal Information for Studies of Extracellular Vesicles; MRD, minimal residual disease; PMA, premarket approval; PSA, prostate-specific antigen; TEPs, tumor-educated platelets.

In clinical practice, liquid biopsy should be viewed as a complementary tool rather than a broad substitute for tissue biopsy. Tissue remains essential for definitive histopathological diagnosis, tumor grading, staging, tumor architecture, immunohistochemistry and assessment of the spatial immune context. For early detection, liquid biopsy should be evaluated using screening-specific endpoints, including sensitivity for stage I–II disease, specificity, positive predictive value, diagnostic workup burden, stage shift and mortality-related outcomes. For patients with established cancer, liquid biopsy may complement tissue biopsy by supporting molecular profiling, treatment selection, treatment-response monitoring, MRD assessment, recurrence surveillance and detection of resistance.

### 2.2 Technical platforms and pre-analytical standardization

Liquid-biopsy detection relies on three broad technical domains: targeted molecular detection, genome-wide or multi-omic profiling, and analyte enrichment. Targeted polymerase chain reaction (PCR)-based assays, including droplet digital PCR (ddPCR), quantitative PCR (qPCR) and quantitative reverse-transcription PCR (qRT-PCR), are useful for predefined DNA or RNA targets because they provide sensitive and quantitative readouts, but they have limited discovery capacity (66–68). Sequencing-based approaches, including next-generation sequencing (NGS), RNA sequencing (RNA-seq), error-corrected targeted sequencing, methylation profiling and shallow whole-genome sequencing (WGS)-based fragmentomics, provide broader genomic, transcriptomic, epigenetic and fragmentomic information, but require higher cost, deeper bioinformatic processing and rigorous error suppression (69–74). For cellular and vesicular analytes, enrichment or isolation methods such as immunomagnetic capture, label-free microfluidics, size-exclusion chromatography and EV immunocapture can support CTC or EV analysis, but each method introduces platform-specific bias in recovery, purity and downstream interpretation (75–78). Representative methods, principles, advantages and limitations are summarized in **Table 3**, and the integrated liquid-biopsy framework linking biofluid sources, analyte classes, analytical interpretation, clinical applications and translational requirements is shown in **Figure 1**.

**Table 3:** Overview of liquid-biopsy detection and analysis techniques for early cancer detection.

Category	Detection/analytical method	Core principle	Typical applications	Advantages	Limitations	Ref
Targeted molecular detection	Targeted PCR-based assays, including ddPCR, qPCR, qRT-PCR and allele-specific PCR	Amplification and quantitative detection of predefined DNA or RNA targets	ctDNA mutations, miRNAs, lncRNAs, circRNAs	Sensitive, quantitative, relatively accessible and suitable for predefined targets	Limited multiplexing; target-dependent; lower discovery capacity	(66–68)
Targeted sequencing	Error-suppressed targeted sequencing, including CAPP-Seq and Safe-SeqS	Target capture, molecular barcoding or consensus sequencing to suppress sequencing errors	Low-frequency ctDNA variants, MRD monitoring, resistance detection	Higher sensitivity than conventional sequencing; suitable for known cancer-associated regions	Costly; requires deep sequencing and bioinformatic expertise; still limited by low tumor fraction	(71)
Genome-wide sequencing	Low-coverage or whole-genome sequencing	Genome-wide profiling of copy-number changes, structural variation and broad cfDNA features	CNVs, broad genomic instability, fragmentomics-compatible analysis	Does not require predefined hotspot mutations; can support broad signal discovery	Lower sensitivity for rare point mutations; computationally intensive; requires large datasets	(30, 69)
Transcriptomic profiling	RNA sequencing and targeted RNA panels	Sequencing or targeted quantification of circulating RNA, EV RNA or platelet RNA	miRNAs, lncRNAs, circRNAs, exosomal RNA, TEP RNA	Captures dynamic gene-expression and host-response signals	RNA degradation, hemolysis and normalization issues; platform variability	(70)
Epigenetic profiling	Methylation profiling, including methylation sequencing and qMSP	Detection of cancer- or tissue-associated DNA methylation patterns	cfDNA methylation, MCED, tissue-of-origin prediction	Tissue-informative; useful for early cancer signals and MCED	Assay-dependent; bisulfite conversion or enrichment bias; requires standardized pipelines	(73, 74)
Fragmentomic profiling	cfDNA fragmentomics / shallow WGS	Analysis of fragment size, end motifs and nucleosome-positioning patterns	cfDNA fragmentomics, tissue-of-origin inference, multi-cancer detection	Genome-wide and complementary to mutation/methylation analysis	Sensitive to pre-analytical variables; cancer-type-dependent; computationally intensive	(30)
Cellular analyte enrichment	CTC enrichment and detection, including immunomagnetic and label-free microfluidic approaches	Enrichment of intact tumor cells based on epithelial markers or physical properties such as size and deformability	CTC enumeration, phenotypic analysis and downstream molecular profiling	Provides intact cellular material and phenotypic information	Rare in early-stage disease; enrichment bias; epithelial-marker dependence; limited standardization	(26, 77, 78)
Vesicular analyte isolation	EV / exosome isolation and characterization, including ultracentrifugation, size-exclusion chromatography, immunocapture and microfluidics	Separation and characterization of vesicles carrying proteins, lipids, DNA and RNA cargo	EV proteins, EV RNA, exosomal immune-checkpoint molecules	Stable cargo; compatible with multi-omics analysis; applicable to multiple biofluids	Variable purity and yield; co-isolation of non-EV particles; lack of universal normalization	(32, 75, 76)
Integrated analysis	Integrated multi-omics / AI-assisted analysis	Computational integration of genomic, epigenomic, transcriptomic, proteomic, fragmentomic, imaging or clinical features	MCED, tissue-of-origin prediction, risk stratification	Combines complementary signals and may improve sensitivity or tissue-of-origin resolution	Missing data, batch effects, feature scaling, overfitting and interpretability challenges	(79)

AI, artificial intelligence; CAPP-Seq, cancer personalized profiling by deep sequencing; cfDNA, cell-free DNA; circRNA, circular RNA; CNV, copy-number variation; CTC, circulating tumor cell; ctDNA, circulating tumor DNA; ddPCR, droplet digital polymerase chain reaction; DNA, deoxyribonucleic acid; EV, extracellular vesicle; lncRNA, long non-coding RNA; MCED, multi-cancer early detection; miRNA, microRNA; MRD, minimal residual disease; qMSP, quantitative methylation-specific polymerase chain reaction; qPCR, quantitative polymerase chain reaction; qRT-PCR, quantitative reverse-transcription polymerase chain reaction; RNA, ribonucleic acid; Safe-SeqS, safe-sequencing system; TEP, tumor-educated platelet; WGS, whole-genome sequencing.

Clinical translation depends not only on analytical sensitivity, but also on the entire implementation pathway. As emphasized by Bao et al., liquid biopsy is moving from a blood-centered concept toward a broader biofluid-based strategy that includes blood, urine, saliva, cerebrospinal fluid and pleural effusions, with applications across early detection, real-time monitoring, MRD assessment and personalized therapy (24). However, broader implementation requires standardized pre-analytical procedures, validated analytical platforms, reproducible bioinformatic pipelines, cost-effective testing models and clinically actionable interpretation frameworks (24).

Recent methodological work further clarifies how standardization should be implemented across the liquid-biopsy workflow. De Rosa et al. proposed a comprehensive methodology for circulating biomarker exploration in peripheral blood, covering PBMCs, CTCs, EVs, circulating tumor RNA, TEPs and circulating cytokines (36). This framework emphasizes that standardization should extend beyond biomarker isolation to include blood collection, processing time, biospecimen fractionation, quality control, normalization, analytical readout and reporting. These requirements are particularly important for early cancer detection, where low-abundance tumor-derived signals are vulnerable to pre-analytical variability, isolation bias and batch effects. Accordingly, CTC-based assays require harmonized enrichment and identification criteria; EV assays require standardized isolation, purity assessment and reporting; TEP- and PBMC-based assays require strict control of handling-related activation artifacts; and circulating RNA assays require careful management of degradation, hemolysis and normalization (32, 36).

### 2.3 Genomic, epigenetic and fragmentomic liquid biopsy

Genomic, epigenetic and fragmentomic cfDNA analyses provide complementary but clinically distinct information. Mutation-based ctDNA testing is relatively mature for tumor genotyping, treatment selection, MRD monitoring and resistance detection in patients with established cancer, whereas its role as a standalone population-level early-screening tool remains limited by low tumor fraction and biological confounders such as clonal hematopoiesis (50, 51). By contrast, cfDNA methylation and fragmentomic patterns may provide broader cancer and tissue-of-origin signals because they capture epigenetic and chromatin-related features rather than only predefined mutations. Hepatocellular carcinoma (HCC) represents a particularly relevant disease context because it often arises in the setting of chronic hepatitis, cirrhosis and inflammation, where conventional surveillance strategies such as ultrasound with or without alpha-fetoprotein (AFP) have imperfect sensitivity. As summarized by Yu and Ma, HCC biomarker discovery is increasingly moving beyond single serum markers toward integrated molecular signatures that may support early detection, personalized treatment selection and prognostic stratification (80). In this context, cfDNA/ctDNA mutation, methylation and fragmentomic analyses provide complementary information but require careful interpretation against the background of chronic liver injury and non-malignant hepatocyte turnover.

#### 2.3.1 ctDNA mutations

ctDNA mutation analysis detects somatic single-nucleotide variants (SNVs), insertions or deletions, copy-number variations (CNVs) and structural variants released from tumor cells. Its strongest clinical value is currently in established cancer, where recurrent driver alterations in genes such as EGFR, KRAS, BRAF, ALK and TP53 can support molecular profiling, targeted therapy selection, MRD assessment and resistance monitoring (33, 81–83). These established applications should be distinguished from early-screening applications, because asymptomatic individuals usually have much lower tumor fractions and unknown tumor genotypes.

For early detection, genome-wide mutation-based and copy-number-based approaches have been explored for pan-cancer signal detection and tissue-of-origin inference. However, their performance depends strongly on tumor fraction, cancer type, stage distribution, sequencing depth, error-suppression strategy and cohort design. Examples such as mutational signature analysis, ctDNA copy-number profiling and integrative frameworks combining methylation and copy-number alterations illustrate the potential of genomic liquid biopsy, but most evidence remains derived from selected or disease-enriched cohorts (84–86). In HCC, low tumor fraction and chronic liver disease further support the need for integrated biomarker frameworks rather than reliance on single genomic features (80).

The major barriers to mutation-based screening are low ctDNA abundance in early-stage disease, sequencing error, clonal hematopoiesis and heterogeneity in pre-analytical and bioinformatic workflows (87–89). Accordingly, mutation-based ctDNA assays should be considered clinically mature mainly for molecular stratification and disease monitoring, but not yet sufficiently sensitive as standalone tests for asymptomatic early cancer screening.

#### 2.3.2 cfDNA methylation

cfDNA methylation is one of the most clinically advanced liquid-biopsy signals for early detection because methylation alterations are frequent, relatively stable and often tissue- or cell-type informative. Unlike mutation-based assays, methylation profiling can capture broad tumor-associated epigenetic changes and may support tissue-of-origin prediction. However, reported performance varies across cancer type, stage composition, control selection, assay design and validation status; therefore, sensitivity, specificity and AUC values should be interpreted as study-specific estimates rather than directly comparable measures of clinical readiness.

Representative methylation biomarkers and clinically translated assays are summarized in **Table 4**. These studies illustrate different levels of maturity. SEPT9 methylation testing has entered clinical use for colorectal cancer screening in defined settings (28, 68), whereas other disease-specific assays, including Bladder EpiCheck, CONFIDENCE, Epi proLung and Lung EpiCheck, are mainly used or evaluated in specific diagnostic or surveillance contexts rather than as broad population-screening tools (91, 92, 94, 102, 103). Recent studies such as MethyDT/IColohunter for colorectal cancer, a multicenter prospective methylation panel for early esophageal cancer and GutSeer/GUIDE for gastrointestinal cancers strengthen the clinical evidence base, but still require careful interpretation according to intended use, population risk and downstream diagnostic pathways (95–97).

**Table 4:** Representative DNA methylation markers for early cancer detection.

Cancer	Product/test name	Study design	Validation status	Case	Marker	AUC	Sensitivity (%)	Specificity (%)	Method	Ref
Bladder	Research	Diagnostic biomarker discovery and validation study using urine samples	Research-stage urinary methylation marker panel; not yet a clinically implemented screening test	134	NRN1, GALR1, HAND2	0.89	76	93	qMSP	(90)
Bladder	Bladder EpiCheck	Diagnostic accuracy and clinical utility study in patients under surveillance for non-muscle-invasive bladder cancer	Clinical decision-support evidence for NMIBC surveillance; not intended for population-level early cancer screening	357	15 proprietary markers	0.961	67.3	88	MSRE-rtPCR	(91)
Bladder	Bladder EpiCheck	Multicenter, prospective, blinded clinical trial in patients under surveillance for non-muscle-invasive bladder cancer	Prospective multicenter validation for NMIBC surveillance; clinically translated urine methylation assay, but not a general early-screening test	353	15 proprietary markers	0.82	68.2	88	MSRE-rtPCR	(92)
Breast	Research	Diagnostic study using plasma cfDNA methylation patterns	Research-stage cfDNA methylation model; small cohort and no population-level prospective screening validation	104	cg23035715, cg16304215, cg20072171, cg21501525	0.9816	89.4	100	qMSP	(93)
Cervical	CONFIDENCE	Subanalysis of an ongoing multicenter TRACE clinical trial in hrHPV-positive women	Clinically relevant triage setting for hrHPV-positive women; based on cervical samples rather than blood and should not be interpreted as MCED	5384	POU4F3 + positive hrHPV test	/	70.1	81.4	rtPCR	(94)
Colorectal	Research / MethyDT	Multicenter, double-blinded, cross-sectional clinical study	Recent multicenter clinical validation of a plasma cfDNA methylation test; promising diagnostic evidence, but not yet evidence of population-level screening outcome benefit	1194	NTMT1, MAP3K14-AS1	0.918	72.7–91.2	85–97.3	qMSP, MethyDT	(95)
Esophageal	Research	Multicenter prospective clinical trial	Prospective multicenter validation for early esophageal cancer detection; stronger evidence than older single-marker studies, but further real-world screening validation is still needed	1429	Septin9, MT-1A, Epo	0.8924	85.5	95.3	qMSP	(96)
Gastrointestinal	GutSeer / GUIDE	Prospective cohort study for blood-based early detection of gastrointestinal cancers	Recent prospective validation of targeted DNA methylation plus fragmentomic sequencing; promising multi-GI cancer detection, but mortality benefit and population-level implementation remain unproven	3328	33032 CpG loci	0.95	82.8	95.8	Targeted DNA methylation	(97)
Liver	Research	Discovery, phase I pilot and phase II clinical validation study	Disease-specific phased validation for HCC detection; relevant to at-risk surveillance populations but not broad population screening	244	HOXA1, EMX1, B3GALT6, ECE1, PFKP, CLEC11A	0.96	95	92	RRBS, qMSP	(98)
Liver	Research	Multicenter case-control biomarker development study using methylated DNA and protein markers	Multi-analyte HCC detection panel with early-stage relevance; requires prospective surveillance validation in at-risk populations	437	HOXA1, EMX1, TSPYL5, B3GALT6 + 2 protein markers	0.92	71	90	rtPCR	(99)
Liver	Research	Large clinical cohort study evaluating ctDNA methylation markers for HCC diagnosis and prognosis	Landmark HCC ctDNA methylation study; supports diagnostic and prognostic value, but not designed as a population-level screening trial	1933	4 CpG loci	0.969	78.6	89.4	ddPCR	(100)
Liver	HepaClear	Blood-based HCC panel development and validation study	Relatively recent HCC-specific methylation plus protein panel; useful for early-stage HCC detection but requires broader prospective validation	60	6 CpG loci	0.918	84.7	92	qMSP	(101)
Lung	Epi proLung	Plasma-based diagnostic validation study distinguishing malignant from non-malignant lung disease	Disease-specific methylation assay for lung cancer diagnosis; not equivalent to population-level LDCT screening or MCED	172	PTGER4, SHOX2	0.88	90	73	rtPCR	(102)
Lung	Lung EpiCheck	Case-control training and validation study in European and Chinese high-risk individuals	International validation of a six-marker plasma methylation assay; promising high-risk diagnostic evidence, but not yet population-level screening validation	367	6 proprietary markers	0.942	84.3	77.7	MSRE-rtPCR	(103)
Multiple	PanSeer	Retrospective analysis of pre-diagnostic plasma samples from a longitudinal cohort plus clinically diagnosed cancer samples	Pre-diagnostic multi-cancer methylation evidence; promising but preliminary, and future prospective longitudinal validation is required	605	10613 proprietary CpG markers	0.97	96	85	WGBS, qMSP	(104)

AUC, area under the receiver operating characteristic curve; cfDNA, cell-free DNA; CpG, cytosine-phosphate-guanine; ctDNA, circulating tumor DNA; ddPCR, droplet digital polymerase chain reaction; GI, gastrointestinal; HCC, hepatocellular carcinoma; hrHPV, high-risk human papillomavirus; LDCT, low-dose computed tomography; MCED, multi-cancer early detection; MSRE-rtPCR, methylation-sensitive restriction enzyme real-time polymerase chain reaction; NMIBC, non-muscle-invasive bladder cancer; qMSP, quantitative methylation-specific polymerase chain reaction; RRBS, reduced representation bisulfite sequencing; rtPCR, real-time polymerase chain reaction; WGBS, whole-genome bisulfite sequencing.

HCC provides a disease-specific example of the translational value and complexity of cfDNA methylation. Aberrant methylation is frequent in hepatocarcinogenesis, and methylation-based plasma assays may complement AFP and imaging-based surveillance in at-risk populations. Landmark and validation studies have supported plasma methylated DNA markers, ctDNA methylation panels and methylated DNA–protein combinations for HCC detection or prognosis (98–100). HepaClear further illustrates the potential of combining methylated CpG sites and protein markers for early-stage HCC detection (101). Together with the HCC biomarker framework summarized by Yu and Ma, these findings support methylation-based liquid biopsy as a disease-relevant strategy for HCC early detection, prognostic assessment and future personalized surveillance, while emphasizing the need to account for chronic liver injury, cirrhosis-related cfDNA release and inflammatory confounding (80).

Methylation-based MCED assays represent a more advanced translational direction because tissue- and cell-type-specific methylation patterns can support both cancer-signal detection and cancer signal origin prediction. Large methylation-based studies such as CCGA and PATHFINDER have demonstrated high specificity and tissue-of-origin potential, but population-level implementation still requires evidence of clinical utility, mortality impact, diagnostic-resolution efficiency, cost-effectiveness and integration with existing screening programs (18, 52, 105). Thus, cfDNA methylation should be considered one of the most promising analyte classes for early cancer detection, but clinical readiness remains indication-specific rather than universal.

#### 2.3.3 cfDNA fragmentomics

cfDNA fragmentomics complements genetic and methylation analysis by examining fragment size distributions, end motifs, nucleosome positioning and chromatin-related fragmentation patterns (106–109). These features may reflect tissue-of-origin, tumor biology, immune-mediated cell death and non-tumor background contributions. Fragmentomic approaches are attractive for early detection because they can use low-coverage WGS, do not require predefined hotspot mutations and may capture cancer-associated chromatin alterations even when ctDNA mutation burden is low (110, 111).

The DELFI (DNA Evaluation of Fragments for Early Interception) approach has shown promising performance across multiple cancers and illustrates the potential of genome-wide fragmentomic profiling (30). In HCC, DELFI-based fragmentomics showed strong sensitivity at high specificity and outperformed AFP in selected settings, supporting its potential relevance in liver cancer surveillance (54). This HCC-specific evidence should be interpreted together with chronic liver disease, cirrhosis-related cfDNA release and inflammatory cell death, which may confound fragmentomic signals; therefore, fragmentomic approaches in HCC should be developed alongside disease-specific clinical variables, AFP or other protein markers and prospective validation in well-defined at-risk cohorts (54, 80).

Recent models have extended cfDNA fragmentomics to lung cancer, ovarian cancer, endometrial cancer and brain tumors, including approaches that integrate fragmentomic profiles with clinical risk factors, protein markers or repeat-element landscapes (111–114). However, performance remains cohort- and cancer-type-dependent. Pre-analytical factors such as blood collection tubes, processing time and sequencing workflow can influence fragmentomic profiles, and biological confounders such as inflammation, tissue injury and non-malignant cell death may mimic or obscure cancer-associated signals (115, 116). Therefore, cfDNA fragmentomics should currently be regarded as an emerging complementary approach rather than a clinically established standalone screening test. Prospective validation in asymptomatic or clinically defined high-risk populations and harmonization of pre-analytical and computational workflows are still required before routine implementation.

#### 2.3.4 Incidental cancer signals from prenatal cfDNA testing

The clinical utility of cfDNA analysis also extends to incidental cancer signal detection during non-invasive prenatal testing (NIPT). NIPT is primarily designed to screen for fetal chromosomal abnormalities, but abnormal maternal cfDNA patterns may occasionally reveal occult malignancy. Because maternal and fetal cfDNA coexist in the maternal circulation, tumor-associated copy-number alterations or abnormal genomic profiles may interfere with fetal aneuploidy interpretation and trigger further maternal evaluation.

Observational studies have shown that non-reportable or unusual NIPT genomic profiles can be associated with subsequent maternal cancer diagnoses, and follow-up strategies such as whole-body magnetic resonance imaging (MRI) and cfDNA sequencing may help localize occult malignancy in selected cases (188, 189). Additional studies in pregnancy-associated cfDNA testing and ovarian cancer demonstrate the biological sensitivity of low-coverage cfDNA sequencing for detecting cancer-associated copy-number alterations (117, 118). However, these findings should be interpreted as incidental or opportunistic cancer signals rather than evidence for an established maternal cancer-screening strategy. Dedicated clinical pathways are needed to manage abnormal cfDNA findings during pregnancy while avoiding unnecessary anxiety, over-investigation and pregnancy-specific harms.

### 2.4 Cellular, vesicular and immune-derived liquid biopsy

#### 2.4.1 Circulating tumor cells

CTCs provide intact cellular material and can capture phenotypic, genomic and functional information that is not available from cfDNA alone, including epithelial-to-mesenchymal transition (EMT) status, metastatic potential and tumor heterogeneity. Experimental evidence suggests that tumor cell intravasation may occur early during tumorigenesis, supporting the biological rationale for CTC-based early detection (119). Several CTC-based assays have been evaluated across tumor types, and representative markers, isolation platforms and diagnostic performances are summarized in **Supplementary Table 1**. Among the most biologically informative observations, CTCs were detected before radiological signs of lung cancer in some high-risk individuals with chronic obstructive pulmonary disease, suggesting that tumor dissemination may precede imaging-detectable lesions (120).

However, CTC-based early detection remains translational rather than clinically established. Reported diagnostic performance varies substantially across cancer type, disease stage, blood volume, enrichment strategy, epithelial-marker dependence and reporting threshold (121–126). A large multicenter validation study reported limited sensitivity for lung cancer detection, underscoring the difficulty of using rare CTCs for population-level screening (127). CellSearch-based CTC enumeration is relatively established for prognostic assessment in selected metastatic cancers, but this application should not be conflated with early cancer screening (26, 27, 33). Overall, CTC assays are biologically informative but currently constrained by low abundance in early-stage disease, enrichment bias, EMT-related marker loss, platform heterogeneity and lack of standardized early-screening thresholds.

#### 2.4.2 Extracellular vesicles and exosomes

EVs and exosomes provide a vesicular layer of liquid biopsy because they carry proteins, lipids, DNA and RNA species that may reflect tumor cells, stromal cells and immune cells. Their stability in plasma, urine, saliva and other biofluids makes them attractive for repeated minimally invasive sampling. EVs and exosomes are incorporated into the vesicular and RNA signal layer of the integrated liquid-biopsy framework shown in **Figure 1**. Reported EV-derived biomarkers for early cancer detection are summarized in **Supplementary Table 2**. Current evidence does not support a universal exosomal marker; instead, the field is moving toward multi-cargo classifiers, EV-proteomics, EV-RNA profiling and AI-assisted high-dimensional pattern recognition (128–132).

From a translational perspective, EV-based assays occupy heterogeneous levels of maturity. Some urine exosomal RNA assays, such as ExoDx Prostate, have been evaluated for prostate biopsy risk stratification and are more clinically mature than most EV-based cancer-detection biomarkers (29). By contrast, most exosomal protein, RNA and multi-omics classifiers remain investigational because diagnostic performance is often derived from retrospective, case-control or platform-specific cohorts. EV-related immune signals are also relevant to treatment-response monitoring. Circulating exosomal programmed death-ligand 1 (PD-L1) has been explored as a dynamic biomarker for response to programmed death 1 (PD-1)/PD-L1 blockade in non-small cell lung cancer (NSCLC), and PBMC-derived exosomes from small cell lung cancer (SCLC) patients receiving chemoimmunotherapy have shown response-associated immune and EMT-related features (133, 134). These findings support the immunological relevance of EV cargo, but they remain preliminary and should be distinguished from population-level early-detection evidence.

The main barriers to EV implementation are variability in isolation, purity assessment, cargo normalization, vesicle subtyping, reporting standards and cross-platform reproducibility. MISEV2023 provides updated recommendations for EV separation, characterization and reporting, and should be treated as a core standardization reference (32). Therefore, EV-based biomarkers should currently be interpreted as promising complementary signals rather than routine standalone screening tools, pending standardized workflows and prospective multicenter validation.

#### 2.4.3 Tumor-educated platelets

TEPs represent a host-response liquid-biopsy substrate because tumor–platelet interactions can remodel platelet RNA, splicing and protein profiles. Platelets are abundant, easily isolated from peripheral blood and may capture systemic tumor–host interactions. TEP-derived RNA signatures have been explored for cancer detection and tumor localization, and representative TEP biomarkers are summarized in **Supplementary Table 3**.

Despite this promise, TEP-based assays remain exploratory to translational. Platelet profiles can be influenced by pre-analytical activation, processing delays, inflammation, thrombosis, comorbidities and sample-handling conditions, which may reduce cancer specificity (135, 136). Low-input sequencing requirements, incomplete workflow standardization, limited availability of specialized platforms and high cost further constrain broad implementation (137–139). Thus, although platelet RNA and protein signatures may support cancer detection and tissue-of-origin inference, TEPs are not yet established for routine screening and require prospective validation in demographically representative cohorts (31).

#### 2.4.4 PBMC-based immune monitoring

PBMC-based monitoring provides an accessible readout of systemic immune status and may complement tumor-derived liquid-biopsy analytes. In lung cancer, PBMCs from NSCLC and SCLC patients receiving chemotherapy and/or anti-PD-L1 therapy showed response-associated immune features, including higher STING and CXCL10 levels in responders and DNA damage response-related alterations associated with clinical response (25). These findings suggest that PBMCs may support chemoimmunotherapy-response monitoring, but PBMC-based biomarkers remain exploratory and require cell-subset-specific analysis, standardized isolation, longitudinal sampling and prospective validation before routine clinical use.

### 2.5 Circulating RNA biomarkers and multi-omics integration

#### 2.5.1 miRNA, lncRNA and circRNA biomarkers

Circulating non-coding RNAs (ncRNAs), including miRNAs, long non-coding RNAs (lncRNAs) and circRNAs, provide dynamic transcriptional and post-transcriptional information relevant to tumor biology and tumor–host interactions. miRNAs are stable in blood and other body fluids, lncRNAs may show tissue- or cancer-context specificity, and circRNAs are structurally resistant to exonuclease degradation. These features make circulating RNA species attractive liquid-biopsy candidates, particularly when detected as cell-free RNAs or EV-associated cargo. However, single RNA markers often lack sufficient cancer specificity because they may also be altered by hemolysis, inflammation, tissue injury and sample-processing variables (57, 58).

Numerous circulating miRNA, lncRNA and circRNA markers have been reported for early cancer detection. Representative miRNA and lncRNA biomarkers are summarized in **Supplementary Table 4**, and representative circRNA biomarkers are summarized in **Supplementary Table 5**. Rather than supporting broad standalone screening use, current evidence suggests that circulating RNA biomarkers are most likely to be clinically useful as components of multi-marker panels, EV cargo classifiers or multi-omics models. For miRNAs, prospectively validated signatures such as the three-miRNA panel for early gastric cancer provide proof of principle, but most reported signatures remain translational and require standardized sampling, RNA extraction, normalization and external validation before routine use (55, 59, 140). For lncRNAs, multi-lncRNA panels have shown diagnostic potential in selected cancers, but many studies remain retrospective, disease-enriched or insufficiently externally validated (141–145). PCA3 represents a more clinically translated RNA example: urinary PCA3, particularly when combined with T2:ERG, has been evaluated in prospective multicenter cohorts and can support prostate biopsy risk stratification (146, 147).

circRNAs add a structurally stable RNA class to liquid biopsy because their closed-loop structure protects them from exonuclease degradation and enables detection in blood, serum, plasma and EV-enriched fractions. Reported circRNA markers and panels have shown diagnostic potential across several cancers, but most studies remain small, retrospective or internally validated. Therefore, circRNA-based assays should not yet be presented as clinically mature screening tools, but rather as promising candidates requiring standardized enrichment, quantification, normalization and multicenter validation. Overall, circulating RNA biomarkers remain exploratory to translational for population-level early detection, and their diagnostic value should be interpreted in the context of intended use, study design, validation status and biological specificity.

#### 2.5.2 Multi-omics fusion strategies

Multi-omics liquid biopsy aims to improve early cancer detection by integrating complementary molecular layers, including genomic, epigenomic, transcriptomic, proteomic and fragmentomic signals. Genomic alterations provide high specificity but may lack sensitivity at very low tumor fraction; methylation and fragmentomic patterns can improve tissue- or cell-of-origin inference; RNA and protein features may reflect functional tumor–host activity; and clinical or imaging variables can provide contextual risk information. By leveraging these complementary layers, multi-omics has the potential to improve sensitivity, specificity, tissue-of-origin resolution and longitudinal monitoring, although the magnitude of benefit depends on assay design, cancer type, disease stage and validation setting (148, 149). Key characteristics of these approaches are summarized in **Supplementary Table 6**.

Recent studies suggest that multi-omics approaches may outperform single-analyte assays in selected settings, including HCC, lung cancer, esophageal cancer and CRC, but this advantage should not be generalized without prospective validation (150–154). Because screening, diagnostic workup, MRD assessment, recurrence surveillance, treatment-response monitoring and prognostic stratification represent distinct intended uses, multi-omics assays should be validated separately for each clinical endpoint rather than assumed to perform similarly across settings.

Computational fusion strategies can be broadly grouped into early, intermediate and late fusion. Early fusion combines low-level features from different modalities before model training, but is sensitive to feature-scale imbalance, missing modalities, batch effects and high-dimensional overfitting. Intermediate fusion learns modality-specific representations before integration and may better preserve modality-specific structure while allowing inter-modality relationships to be modeled. Late fusion combines separate model outputs or risk scores and is often more tolerant of missing modalities, but may miss low-level biological interactions across omics layers (60, 155).

Reliable multi-omics implementation requires careful management of missing data, batch effects, platform-specific noise and feature scaling. These issues may arise from differences in biospecimen collection, storage, library preparation, sequencing depth, methylation platform, mass spectrometry workflow, EV isolation, imaging protocol or computational preprocessing. Complete-case analysis can reduce sample size and introduce selection bias, while poorly scaled features may allow one modality to dominate the model for technical rather than biological reasons. Therefore, modality-specific normalization, batch-effect correction, appropriate imputation, dimensionality reduction and leakage-free cross-validation should be prespecified before model training (61–63).

Despite its promise, multi-omics liquid biopsy remains difficult to implement clinically. High cost, technical complexity, incomplete modality coverage, platform-specific noise and biological confounders, including cfDNA contributions from normal tissues and inflammatory processes, may affect model robustness and clinical interpretation (154, 156, 157). Most multi-omics tests therefore remain in proof-of-concept, retrospective validation or early prospective phases, and should be evaluated according to intended use, reproducibility, diagnostic workup burden, cost-effectiveness and evidence of clinical utility rather than assumed to be ready for routine population-level screening.

### 2.6 Immunological interpretation of liquid-biopsy signals

Liquid-biopsy signals should not be interpreted solely as passive tumor-shedding products. Many circulating analytes also reflect tumor–host immune interactions, including immune surveillance, chronic inflammation, immune escape and treatment-induced immune remodeling. cfDNA and ctDNA may reflect tumor cell death, immune-mediated cytotoxicity and treatment-induced tissue injury; fragmentomic patterns may capture cell-of-origin contributions from tumor and non-tumor cells; EVs can carry immune-regulatory proteins, RNAs and lipids; CTCs may express immune-evasion markers such as PD-L1; and TEPs or PBMC-based signatures may reflect systemic inflammatory and immune activation states rather than purely tumor-intrinsic biology.

This immunological dimension is important because inflammatory and immune-related processes can act as both informative signals and confounders. Chronic inflammation, infection, autoimmune disease, liver injury, smoking-related inflammation, tissue damage and therapy-induced cell death may increase cfDNA release, alter cytokine and chemokine profiles, activate platelets and reshape circulating RNA or EV cargo. These effects may reduce cancer specificity, especially in early-detection settings where tumor-derived signals are low. Therefore, immune-derived and inflammation-associated signals should be interpreted together with clinical context, disease background, sampling time and assay-specific pre-analytical variables.

Exosomal immune checkpoints provide a representative example of liquid-biopsy immunology. Exosomal PD-L1 may suppress antitumor T-cell activity and contribute to systemic immune escape, and circulating exosomal PD-L1 has been associated with disease progression, immune suppression and response or resistance to PD-1/PD-L1 blockade (158). Tumor-derived exosomal programmed death-ligand 2 (PD-L2) has also been reported to impair T-cell function through the PD-1 axis, supporting the concept that EV checkpoint molecules can act as functional immune mediators rather than merely diagnostic cargo.

Immune-derived circulating signals may also support treatment-response monitoring. PBMC-based immune monitoring provides an accessible readout of systemic immune status, and PBMCs from lung cancer patients receiving chemotherapy and/or anti-PD-L1 therapy have shown response-associated immune features, including higher STING and CXCL10 levels in responders and DNA damage response-related alterations associated with clinical response (25). Similarly, PBMC-derived exosomes from SCLC patients receiving chemoimmunotherapy showed response-associated immune- and EMT-related profiles and functional differences between best responders and non-responders (134). These findings support the value of immune-related liquid-biopsy signals for treatment-response monitoring, but they should not be conflated with population-level early cancer screening. Future studies should define whether immune-related signals are intended for early detection, treatment-response assessment, prognosis or mechanistic stratification, because each use case requires distinct validation endpoints.

### 2.7 Comparative clinical maturity of liquid-biopsy analytes

To move beyond a study-by-study catalogue of biomarker performance, liquid-biopsy analytes should be interpreted through a clinically oriented comparative framework. Their value depends not only on analytical sensitivity, but also on intended use, clinical maturity, early-stage sensitivity, tissue-of-origin value, standardization status, study design, validation status and evidence of clinical utility. Screening, diagnosis, MRD monitoring, recurrence surveillance, treatment-response assessment and prognosis should therefore be evaluated as distinct clinical applications rather than as interchangeable endpoints.

In this review, tests with regulatory approval, regulatory clearance or guideline support refer to assays with FDA clearance or approval or explicit inclusion in clinical guidelines for a defined intended use. Clinically validated assays refer to tests evaluated in large clinical cohorts or prospective studies but not yet established as routine standard-of-care screening. Investigational or translational assays have promising analytical or clinical performance but still require external validation, standardized workflows and clinical-utility evidence. Exploratory biomarkers are early-stage candidates supported mainly by discovery, retrospective or small case-control studies.

Clinical maturity should be distinguished from biological promise, and regulatory or guideline status should be interpreted according to the specific intended use. FDA-cleared or FDA-approved assays such as CellSearch-based CTC enumeration, Epi proColon and Shield have defined indications, but these indications are not interchangeable. CellSearch is FDA-cleared for CTC enumeration as an aid in monitoring patients with metastatic breast, colorectal or prostate cancer, whereas Epi proColon and Shield are blood-based CRC screening tests for defined populations. Similarly, guideline-supported urine exosomal RNA testing, such as ExoDx Prostate IntelliScore, supports prostate biopsy risk stratification but should not be described as a general population-level cancer-screening test.

Among genomic and epigenetic analytes, mutation-based ctDNA assays are clinically mature mainly for molecular genotyping, treatment selection, MRD assessment and resistance monitoring in patients with established cancer. Their use as standalone early-screening tools remains constrained by low tumor fraction, clonal hematopoiesis, sequencing error and the need for standardized bioinformatic filtering (50, 51). In contrast, cfDNA methylation is one of the most advanced analyte classes for early detection and MCED because methylation patterns are relatively stable, tissue- or cell-type informative and useful for cancer signal origin prediction. Large methylation-based studies such as CCGA and PATHFINDER support feasibility, but mortality benefit, diagnostic-resolution efficiency, cost-effectiveness and integration with established screening programs remain to be demonstrated (18, 52, 53, 105).

Other analytes are promising but less mature for routine screening. cfDNA fragmentomics provides an orthogonal signal based on fragment size, end motifs and nucleosome positioning, but it remains sensitive to pre-analytical variables, cohort design and biological confounders such as inflammation or tissue injury (30, 54). CTCs provide intact cellular material but are rare in early-stage disease and limited by enrichment bias and platform heterogeneity. EVs and exosomes provide stable multi-cargo signals and may reflect tumor–immune communication, but their implementation depends on standardized isolation, purity assessment, normalization and reporting (32). TEPs, PBMC-based signatures, circRNAs, metabolomic signatures and microbiome-based markers remain largely investigational or exploratory and require prospective validation in representative cohorts (31). Overall, liquid-biopsy analytes should be regarded as complementary rather than interchangeable, and their performance should be interpreted according to regulatory status, guideline support, cancer type, disease stage, study design, validation level and clinical actionability. The key biological characteristics of major liquid-biopsy analytes are summarized in **Table 1**, and their clinical maturity, regulatory status and translational interpretation are summarized in **Table 2**.

## 3 MCED as a screening care pathway

MCED tests are designed for early-detection or screening settings in individuals without a known active cancer diagnosis or in defined high-risk screening populations. This intended use should be distinguished from liquid-biopsy monitoring in patients with established cancer, where serial testing is used for molecular profiling, treatment-response assessment, MRD detection, recurrence surveillance or resistance monitoring. Compared with organ-specific screening, MCED may improve screening efficiency and extend detection to cancers without established population-based screening programs, including pancreatic, ovarian, biliary tract, liver and many upper gastrointestinal cancers (159). However, MCED should be evaluated as a screening care pathway rather than as an isolated blood test.

For population-level screening, MCED performance should not be judged by sensitivity, specificity or AUC alone. Positive predictive value (PPV) and negative predictive value (NPV) are strongly influenced by cancer prevalence, target population risk and screening interval. Because cancer prevalence is low in asymptomatic general populations, even highly specific tests can generate false-positive results that require downstream diagnostic evaluation. Conversely, PPV may be higher in older individuals or clinically defined high-risk groups, but this must be balanced against comorbidity, competing mortality and diagnostic burden. Therefore, MCED implementation should be assessed using population-level endpoints, including PPV, NPV, false-positive workup burden, overdiagnosis, diagnostic-resolution rate, stage shift, late-stage cancer incidence, cancer-specific mortality, quality of life and cost-effectiveness (160–162).

Most MCED platforms currently rely on blood-based analytes, especially cfDNA methylation, ctDNA mutations, fragmentomics, proteins and increasingly multi-analyte or multi-omics combinations. Alternative biofluids, including urine, saliva and cerebrospinal fluid, are being explored for specific tumor types, but blood-based assays remain the most standardized format in MCED development (163–165). cfDNA methylation provides strong tissue-of-origin potential, mutation-based approaches provide specific genomic signals, fragmentomics captures chromatin-related cfDNA patterns and protein or EV-based features may add complementary biological information (29, 30, 49, 52, 166–168). Representative completed MCED clinical studies and trials are summarized in **Table 5**, and additional MCED-related studies are listed in **Supplementary Table 7**.

**Table 5:** Overview of representative completed MCED clinical studies and trials.

Study	Biomarker type	Study design	Sample size	Cancer	Main performance	Validation status	Ref
CancerSEEK	cfDNA mutations + circulating proteins	Case-control development and validation study in patients with non-metastatic, clinically detected cancers and healthy controls	1,005 cancer patients; 812 healthy controls	Eight cancer types	Median sensitivity 70% across eight cancers; specificity >99%; localized cancer to a small number of anatomic sites in most positive cases	Landmark multi-analyte development study; not a population-level screening validation study	(49)
DETECT-A	cfDNA mutations + circulating proteins + PET-CT confirmation	Prospective interventional screening study in women aged 65–75 years without known cancer	10,006	Asymptomatic screening population	Detected 26 cancers first identified by blood testing; specificity 98.9%; PPV 19.4%, increasing to 40.6% when combined with PET-CT	First large prospective interventional MCED screening study; outcome and false-positive follow-up now available	(160, 169, 170)
CCGA targeted methylation validation	cfDNA methylation	Large clinical validation study using independent validation set	15,254 participants across CCGA substudy	>50 cancer types	High specificity and accurate cancer signal origin prediction; sensitivity increased with stage	Landmark methylation-based MCED validation; not a randomized screening-outcome trial	(52)
PATHFINDER	cfDNA methylation	Prospective cohort study of adults aged ≥50 years with and without additional cancer risk	6,662	Outpatient screening setting	PPV 38.0%; specificity 99.1%; many cancers detected in tumor types without USPSTF-recommended screening	Prospective feasibility and diagnostic-resolution study; psychosocial impact data available	(18, 171)
DELFI	cfDNA fragmentomics	Case-control / clinical validation study using genome-wide cfDNA fragmentation profiles	236 cancer patients; 245 healthy controls	Seven cancer types	Overall AUC 0.94; sensitivity 57% to >99% across cancer types at 98% specificity	Landmark fragmentomics study; requires prospective screening validation	(30)
SYMPLIFY	cfDNA methylation	Prospective observational study in symptomatic patients referred from primary care through urgent cancer pathways	5,461	Symptomatic diagnostic pathway	Overall sensitivity 66.3%; specificity 98.4%; PPV increased with extended follow-up	Useful for diagnostic triage in symptomatic pathways; not equivalent to asymptomatic population screening	(172)
SPOT-MAS / K-DETEK	Multimodal ctDNA features, including methylation and fragmentomics	Multicenter prospective clinical validation in asymptomatic adults in Vietnam	9,057 enrolled; 9,024 analyzed	Asymptomatic moderate- to high-risk screening population	Demonstrated feasibility of MCED testing in an Asian population; reported high specificity and clinically structured follow-up	Recent prospective Asian MCED validation; strengthens population diversity evidence	(173, 174)

CCGA, Circulating Cell-free Genome Atlas; cfDNA, cell-free DNA; ctDNA, circulating tumor DNA; DELFI, DNA evaluation of fragments for early interception; MCED, multi-cancer early detection; PET-CT, positron emission tomography–computed tomography; PPV, positive predictive value; SPOT-MAS, Screening for the Presence Of Tumor by Methylation And Size; USPSTF, United States Preventive Services Task Force.

Several prospective or clinically relevant studies illustrate both the promise and the complexity of MCED. In DETECT-A, 10,006 asymptomatic women aged 65–75 years were tested with CancerSEEK, and 26 cancers were first detected by blood testing; specificity was 98.9%, PPV was 19.4%, and PPV increased to 40.6% when combined with positron emission tomography–computed tomography (PET–CT) (169). Subsequent analyses have provided important follow-up information on clinical outcomes and false-positive findings (160, 170). In PATHFINDER, a methylation-based cfDNA test in 6,662 adults aged ≥50 years showed a PPV of 38.0% and specificity of 99.1%, with many detected cancers arising in tumor types without United States Preventive Services Task Force (USPSTF)-recommended screening (18). Recent PATHFINDER data also highlight the importance of psychosocial outcomes after MCED testing (171). THEMIS and the multicenter Asian K-DETEK study further support the feasibility of multimodal cfDNA or multi-omic MCED assays in prospective settings, including detection of stage I–II cancers (173, 174). In symptomatic diagnostic pathways, the UK SYMPLIFY trial enrolled 5,461 patients referred through fast-track National Health Service (NHS) routes and demonstrated that MCED testing may help prioritize imaging and other investigations, although this role should not be equated with asymptomatic population screening (172).

A positive MCED result is not a diagnosis; it initiates a diagnostic-resolution pathway that may include targeted imaging, endoscopy, laboratory testing, tissue biopsy or short-interval follow-up. Therefore, clinical value depends not only on cancer-signal detection, but also on whether downstream procedures can localize cancer efficiently, avoid unnecessary invasive testing and lead to earlier treatment of clinically meaningful cancers. False-positive and indeterminate results may cause anxiety, repeated testing, incidental findings, procedural complications and resource use, whereas false-negative results may provide inappropriate reassurance if their limitations are not clearly communicated. Overdiagnosis remains a concern if screening identifies indolent lesions that would not have caused symptoms or death. Thus, evidence of test accuracy or stage shift alone is insufficient; future trials should evaluate reductions in late-stage cancer incidence, cancer-specific mortality, diagnostic harms and quality of life.

Despite these opportunities, current MCED tests remain incompletely validated for population-level implementation. Most evidence still derives from feasibility, cohort or early implementation studies that report test accuracy, PPV, diagnostic-resolution rate or stage distribution rather than definitive reductions in late-stage incidence or cancer-specific mortality; therefore, survival impact remains uncertain and may be affected by lead-time and length-time bias. Key unresolved issues include optimal testing intervals, integration with established single-cancer screening programs, standardized diagnostic pathways after a positive result and health-system capacity for timely confirmatory imaging, pathology and treatment. Cost-effectiveness is highly sensitive to test price, baseline cancer prevalence, PPV, false-positive burden and downstream diagnostic costs. In parallel, combining MCED outputs with imaging, clinical risk models, ML and AI may help refine risk stratification, but such approaches also require prospective validation before routine population-level use (17, 175).

## 4 AI-assisted interpretation and multimodal implementation

AI can support cancer screening by extracting high-dimensional patterns from imaging, liquid-biopsy, clinical, laboratory and electronic health record (EHR) data. In this context, AI is not a single technology but a group of computational approaches that include ML, DL, natural language processing (NLP) and computer vision (CV). These methods have been explored for image interpretation, biomarker discovery, cancer-signal detection, tissue-of-origin prediction, multimodal fusion, longitudinal risk prediction and workflow triage. Representative AI applications and validation frameworks are summarized in **Table 6**.

**Table 6:** Representative AI applications and validation frameworks for early cancer screening.

AI application area	Cancer	Input data modality	AI task	Validation status	Key limitations	Ref
AI-assisted mammography screening	Breast	Mammography	Screen reading support, lesion detection and workflow triage	MASAI provided randomized screening evidence for AI-supported mammography, and recent multi-institutional studies further support automated mammographic lesion classification	Requires prospective monitoring, population-specific calibration and evaluation of interval cancers, workload, recall rate and equity	(176, 177)
Radiomics and deep-learning CT screening	Lung	LDCT, radiomics and clinical risk factors	Pulmonary nodule detection, malignancy prediction and screening triage	Representative studies support LDCT-based AI and radiomics for lung cancer screening and nodule risk assessment	Generalizability depends on scanner protocol, population risk, smoking distribution, nodule spectrum and external validation	(178, 179)
Liquid-biopsy AI for lung cancer	Lung	cfDNA methylation and fragment-size profiles	Cancer detection and subtype discrimination	Recent cfDNA-based DL models integrate methylation and fragment-size information for lung cancer diagnosis	Early-stage sensitivity, cohort enrichment, sequencing workflow and external validation remain key limitations	(180)
EV-proteomics machine learning	Colorectal	Serum EV-derived proteomic biomarkers	CRC diagnosis and biomarker signature development	Recent ML-based serum EV proteomic study identified and validated diagnostic signatures for CRC	Cost, EV isolation variability and prospective screening validation remain unresolved	(181)
EHR and laboratory-data machine learning	Colorectal	Routine clinical and laboratory data in electronic medical records	Risk stratification and early warning	Recent models support young-onset CRC risk stratification using routine clinical data	Performance depends on data completeness, coding quality, healthcare setting and model calibration	(182)
CT-based deep learning	Colorectal	Contrast-enhanced CT	CRC detection without bowel preparation	Retrospective multicenter evidence suggests feasibility for opportunistic CRC detection	Low PPV in real-world settings, radiation exposure and need for prospective evaluation limit screening use	(183)
MRI-based AI	Prostate	Multiparametric MRI	Clinically significant prostate cancer detection and segmentation	Recent radiology studies support MRI-based DL for assisting clinically significant prostate cancer detection	Requires external validation, scanner harmonization and evaluation of false-positive and false-negative consequences	(184, 185)
PET/CT machine learning	Prostate	68Ga-PSMA-617 PET/CT	Diagnosis and risk assessment	Recent ML models suggest improved diagnosis and risk stratification using PET/CT features	Primarily diagnostic rather than screening; availability, cost and external validation remain limitations	(186)
Clinical and longitudinal biomarker AI	Ovarian	Longitudinal biomarkers, clinical features and pelvic ultrasound	Risk prediction and early-detection support	Recent models and multicenter ultrasound studies support AI-assisted ovarian cancer assessment	Requires prospective validation, subtype-level evaluation and assessment of lead-time benefit	(187–190)
Proteomics and spatial-omics machine learning	HCC	Serum proteomics, spatial omics and clinical data	Early HCC detection and risk stratification	Recent proteomics-driven and spatial-omics-based ML studies support noninvasive early HCC detection	Requires prospective validation in cirrhosis and chronic hepatitis surveillance populations	(191, 192)
AI-enhanced imaging for HCC screening	HCC	Liver ultrasound, MRI and clinical risk factors	Lesion detection, classification and workload reduction	Recent AI-enhanced HCC screening models suggest improved diagnostic accuracy and workload reduction	Sensitivity for small lesions, ultrasound operator dependence and real-world deployment remain concerns	(193)
Metabolomics machine learning	Breast and gastric	Untargeted metabolomics, lipidomics or plasma metabolomics	Biomarker discovery and early-detection model development	Recent metabolomics-ML studies suggest potential for early cancer diagnosis	Requires platform standardization, external validation and assessment of biological specificity	(194, 195)
Multimodal AI fusion	Cross-cancer implementation	Imaging, omics and clinical data	Multimodal fusion, classification and risk prediction	Recent reviews emphasize early, late and hybrid fusion strategies for integrating multi-omics and imaging data	Missing data, batch effects, feature scaling, interpretability and clinical workflow integration remain challenges	(79)
AI reporting and validation framework	Cross-cancer model development	Prediction model data and validation datasets	Transparent reporting, model development and validation	TRIPOD+AI provides updated reporting guidance for prediction models using regression or machine-learning methods	Reporting guidance does not itself establish clinical utility; external validation and calibration remain necessary	(196)
Trustworthy AI deployment and regulatory monitoring	Cross-cancer implementation	AI-enabled clinical tools and medical devices	Lifecycle governance, fairness, robustness, explainability and post-deployment monitoring	FUTURE-AI and recent FDA-device reviews emphasize trustworthy deployment, post-market monitoring and transparency gaps	Algorithm drift, model updating, cybersecurity, accountability and equity require ongoing governance	(197, 198)

AI, artificial intelligence; cfDNA, cell-free DNA; CRC, colorectal cancer; CT, computed tomography; DL, deep learning; EHR, electronic health record; EV, extracellular vesicle; FDA, Food and Drug Administration; HCC, hepatocellular carcinoma; LDCT, low-dose computed tomography; ML, machine learning; MRI, magnetic resonance imaging; PET/CT, positron emission tomography/computed tomography; PPV, positive predictive value; TRIPOD+AI, Transparent Reporting of a multivariable prediction model for Individual Prognosis Or Diagnosis-Artificial Intelligence.

In imaging-based screening, AI has been most extensively studied in mammography, low-dose computed tomography, colonoscopy, digital pathology, prostate magnetic resonance imaging and liver imaging. Randomized or multicenter studies of AI-assisted mammography and lung screening suggest potential benefits for reading efficiency, lesion detection and workflow triage (176–179). However, performance depends strongly on the dataset, cancer prevalence, disease spectrum, imaging protocol, reader workflow and prospective validation setting. Therefore, AI-assisted imaging should be interpreted as decision support within validated screening pathways, rather than as an autonomous replacement for clinicians.

In liquid biopsy and MCED, AI is particularly important because cancer-derived signals are sparse, heterogeneous and embedded within high-dimensional biological and technical background noise. AI models can support mutation filtering, methylation-pattern recognition, fragmentomic feature extraction, protein or EV-based classifier development, multi-analyte fusion and tissue-of-origin prediction. For example, methylation-based MCED models can learn cancer-associated and tissue-specific methylation patterns, whereas fragmentomic models can integrate fragment size, end motifs and nucleosome-positioning features (30, 52). In multi-omics liquid biopsy, AI can further combine genomic, epigenomic, transcriptomic, proteomic, fragmentomic, imaging and clinical variables for risk stratification and cancer signal interpretation (79). These applications are promising, but they should be validated according to intended use, because screening, diagnostic triage, MRD assessment, recurrence surveillance and treatment-response monitoring require different endpoints, thresholds and clinical pathways.

Model development should follow transparent and reproducible standards. High-dimensional radiomics, whole-slide imaging and multi-omics liquid-biopsy models are especially vulnerable to overfitting when the number of extracted features is large relative to the number of cancer events. Internal cross-validation is therefore insufficient for clinical implementation; independent external validation and, ideally, prospective multicenter evaluation are required before clinical deployment. TRIPOD+AI provides updated guidance for transparent reporting of prediction models using regression or machine-learning methods, and PROBAST supports structured assessment of risk of bias and applicability in prediction-model studies (196, 199). Model reports should therefore specify the target population, inclusion and exclusion criteria, preprocessing workflow, feature selection, missing-data handling, training and validation strategy, calibration, subgroup performance and decision-curve or clinical-utility analysis.

Generalizability and fairness are central challenges for AI-enabled cancer screening. Screening populations differ from diagnostic or hospital-enriched datasets because disease prevalence is lower, early-stage lesions are more common and small reductions in specificity can generate large numbers of false-positive results. Cohort bias, spectrum bias and dataset shift may arise from differences in scanners, imaging protocols, staining procedures, sequencing platforms, blood-processing workflows, EHR coding, referral pathways, demographic composition, comorbidity and access to confirmatory care (200, 201). For liquid-biopsy and multi-omics AI systems, pre-analytical variability, batch effects and platform-specific noise may further shift the input distribution and reduce model transportability (202). Therefore, AI models should be evaluated across institutions, geographic regions, technical platforms and clinically relevant subgroups, with reporting of subgroup discrimination, calibration, PPV, NPV, false-positive burden and clinical utility where sample size permits.

Interpretability and workflow integration are also required for safe deployment. Many DL models remain partially opaque, making it difficult to determine whether predictions are driven by biologically meaningful cancer signals or by technical artifacts, site-specific patterns or confounding variables. In screening settings, where AI outputs may trigger imaging, biopsy or surveillance in asymptomatic individuals, uncertainty estimation, failure-mode analysis, human oversight and clear communication of limitations are essential. CONSORT-AI and SPIRIT-AI emphasize transparent reporting of AI interventions in clinical trials and trial protocols, including human–AI interaction and clinical workflow integration (203, 204). The updated CLAIM checklist further supports standardized reporting of AI applications in medical imaging, including input data, preprocessing, model architecture, validation and error analysis (205).

Finally, AI implementation requires lifecycle governance. Models may be affected by temporal changes in population characteristics, clinical practice, data-acquisition protocols and laboratory workflows; therefore, regulatory and post-deployment evaluation should address model updating, algorithm drift, cybersecurity, accountability, auditability and post-market surveillance. A recent review of FDA-approved AI medical devices reported gaps in publicly available information on demographic characteristics, performance-study details, safety data and post-market monitoring, underscoring the need for transparent implementation and ongoing surveillance (198). FUTURE-AI further emphasizes fairness, universality, traceability, usability, robustness and explainability as core principles for trustworthy health AI deployment (197). Overall, AI has shown important methodological progress in cancer screening, especially in imaging-rich domains and multimodal data integration, but its clinical value should be judged not by internal accuracy alone. Until generalizability, calibration, interpretability, workflow compatibility, equity and clinical utility are demonstrated, AI should be used as an assistive technology within validated clinical pathways rather than as an autonomous screening system.

## 5 Regulatory, ethical and health-economic considerations

### 5.1 Regulatory and ethical considerations for liquid biopsy, MCED and AI-enabled screening

Regulatory evaluation of liquid biopsy, MCED and AI-enabled screening should distinguish analytical validity, clinical validity and clinical utility. Analytical validity refers to whether an assay reproducibly and accurately measures the intended biomarker under standardized pre-analytical, analytical and bioinformatic conditions. For liquid biopsy, this includes blood collection, processing time, nucleic-acid or EV isolation, sequencing or assay reproducibility, limit of detection, batch effects, reference materials, quality-control thresholds and reporting standards. Clinical validity refers to whether the test accurately detects cancer, predicts tissue-of-origin or stratifies risk in the intended-use population. Clinical utility refers to whether using the test improves clinical or population outcomes, such as earlier diagnosis of clinically meaningful cancers, reduced late-stage incidence, reduced cancer-specific mortality, improved treatment selection, acceptable diagnostic burden or cost-effectiveness. Therefore, analytical sensitivity, high AUC or technical feasibility should not be interpreted as evidence of clinical utility unless supported by prospective clinical evaluation.

For MCED and population-level screening, ethical concerns arise because testing is performed in asymptomatic individuals and a positive result is not a cancer diagnosis. Uncertain positive results may initiate diagnostic-resolution pathways involving targeted imaging, endoscopy, laboratory testing, biopsy, repeat testing or short-interval follow-up. These pathways may identify clinically meaningful cancers, but they may also generate incidental findings, anxiety, procedural complications, financial burden and health-system opportunity costs. Outcomes following false-positive MCED results have been examined in DETECT-A, supporting the need to evaluate downstream diagnostic pathways rather than test positivity alone (160). Overdiagnosis is another key concern if screening detects indolent lesions that would not have caused symptoms or death. Ethical analyses of MCED and early cancer detection further emphasize uncertainty, false reassurance, overdiagnosis, privacy, justice, medicalization and equitable implementation when screening technologies move into asymptomatic populations (206, 207). Accordingly, MCED implementation should be judged not only by sensitivity, specificity or AUC, but also by PPV, NPV, diagnostic-resolution rate, false-positive workup burden, overdiagnosis, quality of life, late-stage cancer incidence and cancer-specific mortality.

AI-enabled screening tools raise additional regulatory issues because their performance may change after deployment. Unlike static laboratory assays, AI models may be affected by temporal shifts in population characteristics, clinical practice, imaging protocols, sequencing platforms, EHR coding, sample-processing workflows and disease prevalence. Regulatory assessment should therefore address the full lifecycle of the model, including premarket validation, prespecified update procedures, version control, model-change documentation, algorithm drift, cybersecurity, auditability, accountability and post-market surveillance. TRIPOD+AI provides reporting guidance for prediction models using regression or ML methods, including transparent reporting of model development, validation and intended use (196). CLAIM 2024 provides reporting guidance for medical-imaging AI studies, including data partitions, reference standards, model evaluation and reproducibility (205). Recent analyses of FDA-authorized AI devices have identified gaps in public reporting of demographic characteristics, performance-study details, safety data and post-market surveillance, supporting the need for transparent lifecycle monitoring (198).

Privacy and data governance are also central to implementation. Liquid biopsy and MCED assays may generate genomic, epigenomic, fragmentomic, proteomic, imaging and clinical data that can be sensitive and potentially re-identifiable when linked with EHR. Ethical implementation should therefore include informed consent for data generation and secondary use, secure storage, controlled data sharing, transparency about commercial use or algorithm training, and safeguards against discrimination or stigmatization. Incidental germline or cancer-risk findings require clear policies regarding disclosure, confirmatory testing, genetic counseling and family implications. Broader cancer-genomics literature emphasizes that ethical, legal and social issues extend beyond test indication and include privacy, consent, return of results, cultural acceptability, discrimination, family implications and equitable models of care (208).

Equitable access should be treated as a core implementation requirement rather than a secondary concern. Liquid biopsy, MCED and AI-based tools may widen disparities if only well-resourced populations can access testing, confirmatory imaging, biopsy, specialist care or follow-up treatment. Conversely, these technologies could improve equity if they are validated in diverse populations, made affordable, integrated into primary care and accompanied by accessible diagnostic pathways. Recommendations for equitable liquid-biopsy implementation emphasize that adoption barriers should be addressed proactively so that underserved communities are not the last to benefit (209). For MCED specifically, recent work argues that equity should be prioritized from test development through access rather than addressed only after disparities emerge (210). Implementation studies should include underserved, rural, socioeconomically disadvantaged and ethnically diverse populations; report subgroup performance; and evaluate access to confirmatory imaging, pathology and treatment. Equitable implementation will also require insurance coverage or reimbursement strategies, culturally appropriate communication, shared decision-making, community engagement and monitoring for unequal follow-up after positive or indeterminate results (211).

### 5.2 Health-economic evaluation and implementation burden

From a health-system perspective, early cancer detection is not only a clinical strategy but also a major investment decision. Economic evaluations typically use cost–utility analyses based on quality-adjusted life years (QALYs) and incremental cost-effectiveness ratios (ICERs) to compare novel tools, including liquid biopsy, multi-omics panels and AI-enabled strategies, with existing screening or no screening. Relevant costs span the full care pathway: test acquisition, sample processing, confirmatory imaging and biopsy, treatment and surveillance of screen-detected disease, management of false positives and complications, patient time, psychological burden and long-term productivity gains from reduced morbidity and mortality. These elements must be assessed against country-specific willingness-to-pay thresholds, constrained screening budgets and opportunity costs.

Existing economic evidence provides useful principles but should not be overgeneralized. CRC screening with colonoscopy, fecal immunochemical testing and multi-target stool DNA is often cost-effective compared with no screening, highlighting the importance of baseline cancer risk, test performance, adherence, screening interval and downstream treatment costs (212, 213). In HCC, low-cost qMSP-based methylated plasma DNA panels illustrate how assay design can directly influence affordability and scalability in cirrhosis or chronic viral hepatitis surveillance populations, even when formal cost-effectiveness modeling remains limited (214). These examples show that technical performance must be evaluated together with price, infrastructure requirements and target-population risk.

Economic evaluation of MCED is particularly sensitive to assumptions about stage shift, cancer prevalence, PPV, NPV, false-positive rates and the cost and capacity of downstream diagnostic procedures. Modeling studies suggest that cfDNA-based MCED programs could reduce late-stage diagnoses and cancer mortality under certain assumptions, but results depend strongly on test price, screening interval, adherence, diagnostic-resolution efficiency and the cost difference between early- and late-stage treatment (215, 216). Because small specificity losses can generate large numbers of additional imaging tests or biopsies in low-prevalence populations, economic models should incorporate false-positive workup burden, incidental findings, overdiagnosis, patient-reported outcomes and quality of life rather than test cost alone.

AI may improve economic value if it enables risk-adapted screening, reduces unnecessary testing, improves diagnostic triage or optimizes workforce allocation. However, AI-guided strategies introduce additional implementation costs, including data infrastructure, model maintenance, monitoring for algorithm drift, staff training, cybersecurity, liability management and post-deployment auditing. Economic models should therefore incorporate uncertainty around model performance, calibration, subgroup fairness, adherence, implementation capacity and access to confirmatory care. Overall, population-level adoption of liquid biopsy, MCED and AI-enabled screening requires convincing evidence that the full care pathway provides clinical benefit, acceptable harms, equitable access and sustainable economic value.

## 6 Conclusion and perspective

Early cancer detection is increasingly becoming a realistic but complex translational goal that spans tumor biology, assay technology, clinical workflows and health-system implementation (159). The convergence of liquid biopsy, MCED, multi-omics profiling and AI-assisted interpretation is expanding the technical possibilities of cancer screening, but these technologies should not be viewed as replacements for established diagnostic standards. Tissue biopsy and organ-specific imaging remain essential for histopathological confirmation, tumor grading, staging, tumor architecture assessment, immunohistochemistry and evaluation of spatial immune context. The technologies reviewed here should therefore be developed as complementary tools that may expand the window of cancer detection, refine risk stratification and support individualized surveillance when appropriately validated and integrated into clinical pathways (148, 149).

A key translational message from this review is that technical detectability is not equivalent to clinical readiness. Liquid biopsy, MCED, multi-omics profiling and AI should be evaluated according to intended use rather than treated as interchangeable technologies. Screening and MCED applications require evidence based on screening-specific endpoints, including early-stage sensitivity, specificity, PPV, false-positive workup burden, diagnostic-resolution rate, stage shift and outcome benefit. In contrast, diagnostic workup, MRD assessment, recurrence surveillance, treatment-response monitoring and prognostic risk stratification each require different validation endpoints, reporting thresholds and clinical decision pathways.

Liquid-biopsy analytes also differ substantially in clinical maturity. Mutation-based ctDNA assays are already valuable for molecular profiling, treatment selection, MRD assessment and resistance monitoring, whereas their use as standalone early-screening tools remains limited by low tumor fraction and biological confounders such as clonal hematopoiesis. cfDNA methylation and fragmentomics provide broader signals that may support MCED and tissue-of-origin prediction, but their clinical value depends on prospective evidence for stage shift, diagnostic resolution, cost-effectiveness and outcome benefit. CTCs, EVs, circulating non-coding RNAs, TEPs, PBMC-based immune signatures, metabolomic markers and microbiome-based markers provide important biological and immunological information, but most applications remain translational or exploratory and require assay standardization, reproducibility testing and multicenter validation.

From an immunological perspective, liquid-biopsy immune signals may be most clinically useful when they are linked to specific cancer-immunity and immunotherapy decisions. PBMC-based signatures, immune-cell-derived EVs, exosomal PD-L1/PD-L2, cytokine- or proteomic immune markers, TEP profiles and immune-related cfDNA or fragmentomic patterns may help characterize systemic immune activation, immune suppression, tumor–immune crosstalk and treatment-induced immune remodeling (25, 133, 134, 158). In patients receiving immunotherapy or chemoimmunotherapy, these signals may support patient stratification, early response assessment, detection of emerging resistance and longitudinal monitoring of immune escape (25, 133, 134). However, they should complement rather than replace established tissue-based immune biomarkers such as immunohistochemistry-based PD-L1 assessment, tumor immune-contexture evaluation and other clinically validated markers. Future studies should therefore validate immune-related liquid-biopsy readouts against defined immunotherapy endpoints, including objective response, durable benefit, immune-related adverse events, progression-free survival and overall survival, while accounting for inflammatory confounders and treatment timing.

For MCED and AI-enabled screening, the critical translational question is no longer only whether weak cancer signals can be detected, but whether testing can be implemented as a safe, equitable and cost-effective care pathway. MCED should be evaluated through PPV, NPV, diagnostic-resolution pathways, false-positive workup burden, overdiagnosis, quality of life, late-stage cancer incidence and cancer-specific mortality, rather than as an isolated laboratory test. Similarly, AI should be used as assistive decision support, with performance judged by generalizability, calibration, interpretability, fairness, workflow compatibility, post-deployment monitoring and demonstrated clinical utility rather than internal accuracy alone.

Future research should move from biomarker discovery toward implementation-oriented validation. Priority areas include intended-use-specific endpoints, representative populations, standardized pre-analytical and analytical workflows, diagnostic-resolution pathways, health-economic evaluation, equity assessment and regulatory oversight. Prospective, multicenter and demographically representative studies will be essential to determine where liquid biopsy, MCED, multi-omics and AI provide meaningful benefit beyond existing screening and diagnostic approaches. The most realistic translational path is an evidence-based, risk-adapted model in which these technologies complement current care, prioritize individuals most likely to benefit and guide efficient downstream evaluation. If these requirements are met, they may contribute to earlier diagnosis, more precise surveillance and more equitable reductions in cancer burden.
