# Supplementary material for: Liquid biopsy, multi-cancer early detection, and artificial intelligence: new frontiers in cancer screening from a technological and immunological perspective
Source: Front Immunol. 2026 Jun 16;17:1854718. doi: 10.3389/fimmu.2026.1854718 (PMC13314776; doi:10.3389/fimmu.2026.1854718)
Supplement: Supplementary file 2 [file Table1.docx]

**Liquid biopsy, multi-cancer early detection, and artificial intelligence: New frontiers in cancer screening from a technological and immunological perspective**

Dong Wu^1#^, Qing Yu^2#^, Qixiang Wu^3^, Xiaoying Wang^1^, Haojie Wei^1^, Fengli Li^4^, Guangli Li^1^, Xiaowu Wang^5*^, Xiaojuan Wang^1*^

*1 Department of Clinical Pharmacy, Fuyang People's Hospital, Fuyang, Anhui, 236000, China*

*2 Department of Pharmacy, Tongling People's Hospital, Tongling Academy of Medical Sciences, Tongling, Anhui, 244000, China*

*3 Inflammation and Immune Mediated Diseases Laboratory of Anhui Province; School of Pharmaceutical Sciences, Anhui Medical University, Hefei, Anhui, 230032, China*

*4 Department of Clinical Pharmacy, Fuyang Cancer Hospital, Fuyang, Anhui, 236000, China,*

*5 Department of* *Clinical Laboratory, The Second People's Hospital of Fuyang City, Fuyang Infectious Disease Clinical College of Anhui Medical University, Fuyang, Anhui, 236000, China*

# Authors contributed equally to this work.

*Corresponding Authors:

Xiaojuan Wang

*Department of Clinical Pharmacy, Fuyang People's Hospital, Fuyang, Anhui, 236000, China*

E-mail: wxj0037@163.com

Xiaowu Wang

*Department of Clinical Laboratory, The Second People's Hospital of Fuyang City, Fuyang Infectious Disease Clinical College of Anhui Medical University, Fuyang, Anhui, 236000, China*

E-mail: [wangxiaowu19880218@126.com](mailto:wangxiaowu19880218@126.com)

**Supplementary Table 1** Representative CTC markers for early cancer detection

| Cancer | Study design | Validation status | Case | CTC isolation technology | AUC | Sensitivity (%) | Specificity (%) | Results | Ref |
| --- | --- | --- | --- | --- | --- | --- | --- | --- | --- |
| Breast | Case-control study | Internal comparative evaluation | 343 | CytoSorter | 0.856 | 77.5 | 93.8 | The detection rates of CTC for healthy volunteers, and patients with BBD and breast cancer were 17.2%, 40.7%, and91.2%, respectively. For participants with clinically diagnosed BC, CTC detection rates in stage I–III BC patients were 92.9%, 87.2%, and 100%, respectively. | 1 |
| Breast | Case-control study | Proof-of-concept | 58 | Nanostructured titanium oxide coated slides to capture all cells post  erythrolys | / | 75 | 97 | CTCs were detected in 57% (16/28) of breast cancer patients; among these, 18% (5/28) had detectable CTC clusters. 3% (1/30) of healthy donors had detectable CTCs. | 2 |
| Colorectal | Prospective diagnostic case-control study | Internal clinical validation | 667 | CellMax | 0.94 | 95.2 | / | The AUC for the detection of colorectal cancer was 0.94 with a sensitivity of 95.2% and the AUC for adenomas was 0.868. | 3 |
| Gastric | Prospective case-control study | Single-center internal validation | 147 | FAST disc | / | 85.3 | 90.3 | CTCs were detected in 90.5% (105/116) of gastric cancer patients and in 9.68% (3/31) of donors | 4 |
| HCC | Diagnostic case-control study | Same-cohort diagnostic validation | 176 | CanPatrol™ | 0.774 | 50.44-72.6 | 61.4-92.9 | The AUC values for total CTCs, AFP, and a combined model were 0.774, 0.669 , and 0.821. | 5 |
| HCC | Prospective case-control study | Pilot validation | 69 | NanoVelcro CTC chip | 0.92 | 84.2 | 88.5 | HCC-CTCs were detected in  97% (59/61) HCC patients and in 25% (2/8) healthy controls | 6 |
| Lung | Prospective cohort study | Proof-of-concept | 245 | ISET | / | / | / | CTCs were detected in 3% (5/168) COPD patients and all development lung nodules with 1-4 years | 7 |
| Lung | Prospective multi-center cohort study | Prospective multicenter validation | 614 | ISET | / | 26.3 | / | At baseline, the sensitivity of CTC detection for lung cancer detection was 26.3%. ISET was unable to predict lung cancer or extrapulmonary cancer development. | 8 |
| Multiple | Observational cohort study | Observational | 542 | ISET | / | / | / | CTCs were detected in 100% (277/277) of known cancer patients and in 50% (132/265) of asymptomatic individuals during screening. Subsequent follow-up tests, conducted within 1-10 months of a positive CTC result, identified early cancerous lesions in 20% (24/132) of these screen-positive individuals. | 9 |
| NSCLC | Case-control study / cross-sectional observational study | Same-cohort assay evaluation | 309 | ISET | / | / | / | CTCs were detected in 41% (102/250) of NSCLC patients, and circulating non-haematological cells were detected in 0% (0/59) of healthy volunteers | 10 |
| PDAC | Prospective observational study | Single-center prospective internal validation | 100 | EpCAM-based NanoVelcro CTC chip | 0.867 | 75 | 96.4 | CTCs were detected in 75% (54/72) of PDAC patients and in 3.6% (1/28) of non-PDAC patients. | 11 |
| Prostate | Prospective diagnostic study | Internal model validation | 265 | Parsortix PC1 system | 0.707 | 51.8 | 80.3 | A positive CTCs score was found in 54% (84/155) PC patients | 12 |
| HCC, hepatocellular carcinoma; NSCLC, non-small cell lung cancer; PDAC, pancreatic ductal adenocarcinoma; CTCs, circulating tumor cells; BBD, benign breast disease; AUC, area under the curve; AFP, alpha-fetoprotein; COPD, chronic obstructive pulmonary disease; ISET, isolation by size of epithelial tumor cells | | | | | | | | | |

**Supplementary Table 2.** Representative exosomal and extracellular-vesicle markers for early cancer detection

| Cancer | Study design | Validation status | Case | Exosomes | Fluid | Cargo type | Major method | AUC | Sensitivity (%) | Specificity (%) | Ref |
| --- | --- | --- | --- | --- | --- | --- | --- | --- | --- | --- | --- |
| Breast | Case-control study | Cross-validation | 38 | miR-375, PD-L1 mRNA | Serum | miRNA | qRT-PCR | / | / | / | 13 |
| Breast | Case-control study | Two-cohort validation | 562 | Del-1 | Plasma | Protein | LC-MS/MS | 0.961 | 94.7 | 86.36 | 14 |
| Colorectal | Case-control study | Internal train | 100 | miR-23a-3p, miR-92a-3p, miR-125a-3p and miR-150-5p | Plasma | miRNA | qRT-PCR | 0.986 | 91.67 | 100 | 15 |
| Colorectal | Case-control study | validation cohorts | 1179 | Seven lncRNA | serum | lncRNA | qRT-PCR | 0.810 | 76 | 71 | 16 |
| Colorectal | Case-control study | ROC only | 135 | miR-126, miR-1290, miR-23a, miR-940 | serum | miRNA | qRT-PCR | 0.95 | 83.3-89.9 | 71.4-85.7 | 17 |
| Colorectal | Diagnostic cohort study | Independent validation | 30 | Panel of 10 protein markers | Plasma | Protein | DIA-MS, ML | 1.00 | / | / | 18 |
| Colorectal | Case-control study | Independent validation | 184 | 10 RNA markers | plasma | miRNA/lncRNA | RNA sequencing, qRT-PCR | 0.87 | 99 | 79.3 | 19 |
| Colorectal | Case-control study | Internal validation | 142 | miR-320d | Serum | miRNA | qRT-PCR | 0.633 | 62 | 64.7 | 20 |
| Colorectal | Case-control study | Independent validation | 215 | hsa-circ-0004771 | Serum | circRNAs | qRT-PCR | 0.59 | 54.29 | 68.57 | 21 |
| Gastric | Case-control study | Internal validation | 246 | HOTTP | Serum | lncRNA | qRT-PCR | 0.827 | 69.8 | 85 | 22 |
| Gastric | Case-control study | Same-cohort validation | 1468 | GKN1 | Serum | Protein | ELISA | 0.9954 | 91.2 | 96 | 23 |
| Gastric | Case-control study | Training/validation | 169 | piR-1029, piR-15254, novel-piR-35395, novel-piR-32132, novel-piR-43597 | Serum | piRNA | sRNA-seq, qRT-PCR | 0.981 | / | / | 24 |
| Glioma | Diagnostic cohort study | Independent validation | 60 | miR-17-5p, miR-21-5p, miR-27a-3p, LOX, SLCO3A1 | Plasma | miRNA | qRT-PCR, RNA-seq | 0.854 | / | / | 25 |
| HCC | Case-control study | Internal validation | 249 | lncRNA THEMIS2-211, PRKACA-202, H19-204 | Plasma | lncRNA | qRT-PCR | 0.887 | 41.8-83.6 | 75.7-97.3 | 26 |
| HCC | Case-control study | Independent validation | 12 | lncRNA LUCAT-1, EGFR-AS1 | Plasma | lncRNA | qRT-PCR | 0.830-0.895 | / | / | 27 |
| HCC | Case-control study | Clinical validation | 29 | GPC1, EphA2 | Plasma | Protein | ICP-MS | 1.00 | 100 | 100 | 28 |
| Liver | Case-control study | Validation cohort | 178 | miR-215-5p | Serum | miRNA | qRT-PCR | 0.934 | 90.7 | 75 | 29 |
| Lung | Case-control study | Cohort validation | 126 | miR-200b-3p,miR-3124-5p, miR-92b-5p | serum | miRNA | qRT-PCR | 0.93 | / | / | 30 |
| Lung | Retrospective/prospective study | Retrospective/prospective | 122 | Autoantibody panel on sEVs (P53, PGP9.5, SOX2, GAGE7, GBU4-5, MAGEA1, CAGE) | Plasma | Protein | ELISA, AUC analysis | 0.8007 | / | / | 31 |
| Multi-cancer types | Retrospective study | Test-set validation | 278 | CD9, CD63, CD81, TSG191 | Plasma | Protein | Surface-enhanced Raman spectroscopy with AI | 0.97 | 89.4 | 96.3 | 32 |
| HCC, hepatocellular carcinoma; lncRNA, long non-coding RNA; miRNA, microRNA; circRNAs, circular RNAs; piRNA, PIWI-interacting RNA; qRT-PCR, quantitative reverse transcription polymerase chain reaction; LC-MS/MS, liquid chromatography-tandem mass spectrometry; ELISA, enzyme-linked immunosorbent assay; DIA-MS, data-independent acquisition mass spectrometry; ML: machine learning; RNA-seq, RNA sequencing; qPCR, quantitative polymerase chain reaction | | | | | | | | | | | |

**Supplementary Table 3.** Representative tumor-educated platelet markers for early cancer detection

| Cancer | Study design | Validation status | Case | Marker | AUC | Sensitivity (%) | Specificity (%) | Biosample | Method | Ref |
| --- | --- | --- | --- | --- | --- | --- | --- | --- | --- | --- |
| Multi-cance | Retrospective bioinformatic diagnostic study | Internal validation by held-out test set | 2018 | SLC38A2, DHCR7, IFITM3, USF3/KIAA2018, ARL2, DSTN | 0.928 | 91.74 | 67.95 | Platelet | RNA-seq | 33 |
| Breast | Case-control study | Candidate biomarker verified by qRT-PCR | 703 | TPM3 mRNA | 0.84 | / | / | Platelet | RNA-seq, qRT-PCR | 34 |
| Colorectal | Case-control diagnostic study | Internal algorithmic validation | 283 | mRNA | 0.986 | 97 | 96 | Platelet | RNA-seq | 35 |
| Colorectal | Case-control diagnostic study | Internally validated diagnostic model | 1628 | mRNA | 0.91 | / | / | Platelet | RNA-seq | 36 |
| Colorectal | Retrospective cohort study | Internal validation and external validation set | 322 | mRNA | 0.92 | 0.885 | 0.868 | Platelet | RNA-seq | 37 |
| Glioblastoma | Case-control diagnostic study | Independent validation series | 347 | TEPs-derived RNA | 0.97 | / | / | Platelet | RNA-seq | 38 |
| Lung | Case-control diagnostic study | Discovery cohort | 142 | AHNAK mRNA | 0.78 | / | / | Platelet | RNA-seq | 39 |
| Lung | Case-control diagnostic study | qPCR validation in a large clinical cohort | 629 | linc-GTF2H2-1, RP3-466P17.2, lnc-ST8SIA4-12 | 0.921 | 82.6 | 87.1 | Platelet | qRT-PCR | 40 |
| NSCLC | Retrospective case-control study | Leave-one-out cross-validation | 633 | 48-gene TEP | 0.76 | 92.5 | 82.7 | Platelet | RNA-seq | 41 |
| NSCLC | Case-control diagnostic study | Test cohort and blinded validation cohort | 534 | ITGA2B | 0.922 | 92.8 | 78.6 | Platelet | RNA-seq, qRT-PCR, ddPCR | 42 |
| NSCLC | Retrospective case-control diagnostic study | qPCR-based clinical validation | 290 | SNORD55 | 0.803 | 79.3 | 68.3 | Platelet | qRT-PCR | 43 |
| NSCLC | Case-control diagnostic study | Large validation cohorts | 779 | RNA | 0.89 | / | / | Platelet | thromboSeq | 44 |
| Sarcoma | Proof-of-concept case-control diagnostic study | Training, evaluation, and independent validation series | 160 | mRNA | 0.93 | 88 | 86 | Platelet | thromboSeq | 45 |
| NSCLC, non-small cell lung cancer; RNA-seq, RNA sequencing; qRT-PCR, quantitative reverse transcription polymerase chain reaction; ddPCR, droplet digital polymerase chain reaction | | | | | | | | | | |

**Supplementary Table 4.** Representative miRNA and lncRNA markers for early cancer detection

| Cancer | Study design | Validation status | Case | Marker | AUC | Sensitivity (%) | Specificity (%) | Biosample | Ref |
| --- | --- | --- | --- | --- | --- | --- | --- | --- | --- |
| Bladder | Case-control diagnostic study | Compared with qRT-PCR in the same clinical urine sample set | 220 | lncRNA-UCA1 | 0.966 | 89.2 | 93.3 | Urine | 46 |
| Esophageal | Case-control diagnostic study | Training set plus validation set | 148 | 6-miRNA panel: hsa-miR-21-5p, hsa-miR-93-5p, hsa-miR-181a-5p, hsa-miR-17-5p, hsa-miR-25-3p, hsa-miR-151a-3p | 1.000 | 100.0 | 100.0 | Serum | 47 |
| Breast | Case-control diagnostic study | Training/testing phases plus independent external validation | 806 | miR-106a-3p, miR-106a-5p, miR-20b-5p, miR-92a-2-5p | 0.889 | 82 | 79 | Serum exosome | 48 |
| Breast | Case-control observational study | Single-cohort clinicopathologic analysis | 200 | lncRNA HIT | 0.75 | 80 | 50 | Plasma | 49 |
| Colorectal | Mechanistic experimental study | Functional validation in vitro/in vivo | 240 | miR-196b-5p | 0.88 | / | / | Serum exosome | 50 |
| Glioblastoma | Case-control diagnostic study | Three discovery cohorts confirmed in two validation cohorts | 195 | nine miRNA | 0.83 | 28 | 95 | CSF | 51 |
| HCC | Case-control study | 10-fold cross-validation | Study1:100  Study2: 70  Study 3:100 | Study 1: miRNA-483-5p;  Study 2: miRNA-21;  Study 3: miRNA-155 | Study 1: 0.9890; Study 2: 0.9963; Study 3: 0.7993 | Study 1: 98.0; Study 2: 97.78; Study 3: 83.2 | Study 1: 99.0; Study 2: 98.89; Study 3: 95.8 | Serum | 52 |
| HCC | Retrospective case-control diagnostic study | TCGA analysis plus single-center qRT-PCR clinical confirmation | 130 | lncRNA CASC9 | 0.933 | 87.5 | 90 | Plasma | 53 |
| LSCC | Case-control diagnostic study | Plasma–tissue qRT-PCR comparison within a single cohort | 840 | miR-155 | 0.757 | 58.4 | 69.5 | Plasma | 54 |
| Lung | Retrospective case-control diagnostic study | Internal training/validation | 499 | LINC00312 | 0.819 | 70.1 | 96.3 | Serum exosome | 55 |
| Prostate | Prospective multi-center diagnostic study | Separate multicenter validation cohort | 516 | PCA3 lncRNA | / | 90.5 | 32.2 | Urine | 56 |
| RCC | Case-control diagnostic study | Database screening plus clinical training and internal validation | 209 | miR-21-5p, miR-150-5p, miR-145-5p, miR-146a-5p | 0.938 | 90.79 | 93.75 | Serum | 57 |
| Thyroid | Prospective case-control diagnostic study | Single-cohort qRT-PCR and prognostic analysis | 219 | miR-29a | 0.884 | 85.7 | 78.9 | Serum exosome | 58 |
| uroepithelial carcinoma | Prospective case-control diagnostic validation study | Training/validation split with cross-validation | 178 | miR-1343-5p, miR-6087 | 0.76-0.87 | 59-83 | 68-84 | Serum | 59 |
| PCNSL, primary central nervous system lymphoma; CSF, cerebrospinal fluid; HCC, hepatocellular carcinoma; LSCC, laryngeal squamous cell carcinoma; RCC, Renal cell carcinoma; qRT-PCR, quantitative reverse transcription polymerase chain reaction; TCGA, the cancer genome atlas; lncRNA, long non-coding RNA; miRNA, microRNA; | | | | | | | | | |

**Supplementary Table 5.** CircRNAs as diagnostic biomarkers for cancer

| Cancer type | Study design | Validation status | Case | Marker | AUC | Sensitivity (%) | Specificity (%) | Biosample | Ref |
| --- | --- | --- | --- | --- | --- | --- | --- | --- | --- |
| Thyroid | case-control diagnostic study | qRT-PCR validation in two independent clinical cohorts | 304 | circRAPGEF5, hsa_circ_0058124 | 0.8 | 67.4-86.7 | 61.8-88.4 | Serum | 60 |
| PDA | Multi-phase case-control diagnostic study | Training cohort | 151 | 5-circRNA plasma panel | 0.81 | / | / | Plasma | 61 |
| Biliary Tract | Case-control diagnostic study | Clinical validation | 70 | 4-circRNA panel | 0.83 | / | / | Plasma | 62 |
| Thyroid | Case-control diagnostic study | Sequencing-based screening plus qRT-PCR and functional validation | 130 | Hsa_circ_0124055, Hsa_circ_0101622 | 0.911 | 71.2-89.4 | 81.8-93.9 | Serum | 63 |
| Breast | Case-control diagnostic study | Microarray discovery and qRT-PCR confirmation in clinical plasma samples | 522 | hsa_circ_0008673 | 0.833 | 55 | 97.1 | Serum | 64 |
| Breast | Case-control diagnostic study | Training set and test set validation | 414 | hsa_circ_0000091, hsa_circ_0067772, hsa_circ_0000512 | 0.974 | 66.7-91.2 | 52.9-80.4 | Serum | 65 |
| Breast | Case-control diagnostic study | Same-cohort qRT-PCR validation | 190 | hsa_circ_0000615 | 0.904 | 76.8 | 88.4 | Serum | 66 |
| Breast | Prospective case-control diagnostic classifier study | Training cohort and validation cohort | 289 | five circRNA | 0.83 | 74.4-89.1 | 70.0-85.3 | Serum | 67 |
| Gastric | Case-control diagnostic study | Clinical validation | 349 | hsa_circ_0032777 | 0.787 | 81.1 | 56 | Serum | 68 |
| Gastric | Case-control diagnostic study | Plasma expression validation | 229 | hsa_circ_0002762 | 0.784 | 90.6 | 53.8 | Serum | 69 |
| Gastric | Case-control diagnostic study | Training cohort and validation cohort | 288 | eight circRNA | 0.83 | 89 | 62 | Serum | 70 |
| Gastric | Case-control diagnostic study | qRT-PCR methodological validation | 320 | circ_0004771 | 0.831 | 67.5 | 79.17 | Serum | 71 |
| Gastric | Case-control mechanistic study | Functional validation | 255 | circTMC5 | 0.821 | 61.9 | 92 | Serum | 72 |
| Gastric | Case-control diagnostic study | Same-cohort qRT-PCR and functional EMT-pathway validation | 294 | circPTPN22 | 0.857 | 78 | 87 | Serum | 73 |
| Gastric | Case-control diagnostic study | qRT-PCR validation | 226 | hsa_circ_0000702 | 0.745 | 82.69 | 48.44 | Serum | 74 |
| Gastric | Case-control diagnostic study | qRT-PCR validation | 192 | Hsa_circ_0065149 | 0.769 | 79.2 | 61.5 | Serum | 75 |
| Esophagus | Case-control study | Profiling and clinical association analysis | 100 | hsa_circ_0001946, hsa_circ_0043603 | 0.928 | 84 | 98 | Serum | 76 |
| Prostate | Diagnostic classifier development and validation study | Training cohort plus two independent validation cohorts | 1002 | circPDLIM5, circSCAF8, circPLXDC2, circSCAMP1, circCCNT2 | 0.82 | 86.11 | / | urinary | 77 |
| Oral squamous cell carcinoma | Case-control diagnostic study | Microarray screening followed by qRT-PCR validation | 93 | hsa_circ_0001874, hsa_circ_0001971 | 0.922 | 59.38 | 81.25 | saliva | 78 |
| Pancreatic | Case-control diagnostic biomarker study | qRT-PCR validation | 124 | hsa_circ_0001666, hsa_circ_0006220 | 0.884 | 77.42-96.77 | 72.58-51.61 | Serum exosome | 79 |
| PDA, Pancreatic Ductal Adenocarcinoma; qRT-PCR, quantitative reverse transcription polymerase chain reaction; ddPCR, droplet digital polymerase chain reaction; EMT, epithelial-mesenchymal transition | | | | | | | | | |

**Supplementary Table 6.** Technical characteristics and applications of multi-omics in liquid biopsy

| Omics Layer | Typical Analyte in Liquid Biopsy | Platform / Methods | Primary Advantage | Limitation | Ref |
| --- | --- | --- | --- | --- | --- |
| Genomics | ctDNA mutations, CNVs, fusions | NGS (targeted sequencing, WGS/WES) | High specificity for tumor presence | Low sensitivity in early-stage; CHIP confounders | 80 |
| Epigenomics | DNA methylation patterns, nucleosome footprints | WGBS, bisulfite sequencing, methylation arrays | Early tumor-derived signal; tissue origin | Requires high coverage; assay variability | 81, 82 |
| Transcriptomics | cfRNA (mRNA, miRNA, lncRNA) | RNA-seq, qPCR panels, exosome RNA profiling | Reflects gene expression dynamics | RNA unstable in plasma; signal dilution | 83-86 |
| Proteomics | Circulating tumor proteins, exosome proteins | Mass spectrometry, immunoassays | Functional tumor output; host response | Complexity, low abundance, cost | 87-90 |
| Fragmentomics | DNA fragment size, end motif, methylation + fragment pattern | Low-coverage WGS, computational models | Orthogonal tumor signature | Analytical complexity; still early in translation | 91 |
| ctDNA,circulating tumor DNA; CNVs, copy number variations; NGS, next-generation sequencing; WGS, whole-genome sequencing; WES, whole-exome sequencing; CHIP, clonal hematopoiesis of indeterminate potential; WGBS, whole-genome bisulfite sequencing; cfRNA, cell-free RNA; mRNA, messenger RNA; miRNA, microRNA; lncRNA, long non-coding RNA; RNA-seq, RNA sequencing; qPCR, quantitative polymerase chain reaction | | | | | |

**Supplementary Table 7.** Representative MCED clinical trials

| Study/Assay ID | No. of cancer | Study design | Case | Type of biomarkers | Positive rate (%) | Sensitivity (%) | Specificity (%) | PPV (%) | TOO  (%) | Ref |
| --- | --- | --- | --- | --- | --- | --- | --- | --- | --- | --- |
| CancerSEEK | 8 | Case-control study | 1005 | Circulating proteins, ctDNA mutations | healthy controls: 0.86 | 62 | >99 | / | 63 | 92 |
| CCGA | >50 | Case-control study | 15000 | ctDNA methylation | / | 67.3 | 99.3 | 51 | 93 | 93 |
| DELFI | 7 | Case-control study | 423 | ctDNA fragmentation | / | 73 | 98 | / | 61 | 94 |
| DETECT-A | 8 | Prospective interventional study | 10006 | Circulating proteins, ctDNA mutations | 1.35 | 27.1 | 98.9 | 19.4 | / | 95 |
| Exosome-SERS-AI | 6 | Case-control study | 278 | Exosome | / | 90.2 | 94.4 | / | 72 | 32 |
| exoRNA-MCED | 8 | Multi-stage | 1385 | Exosome | / | / | / | / | / | 96 |
| GutSeer | 5 | Case-control study | 1844 | ctDNA methylation + fragmentation | / | 86.2 | 96.7 | / | 82 | 97 |
| K-DETEK | 5 | Multicenter prospective study | 9024 | ctDNA methylation, fragmentation, copy number | 0.48 | 70.83 | 99.71 | 39.53 | 52.94 | 98 |
| MERCURY/CanScan (Jinling) | 13 | Case-control study | 3724 | ctDNA fragmentation | / | 53.5 | 98.1 | 25 | 63.2 | 99 |
| OncoSeeK | 9 | Case-control study | 7565 | Circulating proteins | / | 51.7 | 92.9 | 65.8 | 66.8 | 100 |
| PanSeer | 5 | Case-control study | 1246 | ctDNA methylation | / | 87.6 | 96.1 | / | / | 101 |
| PATHFINDER | >50 | Prospective cohort study | 6662 | ctDNA methylation | 1.4 | 28.9 | 99.1 | 38 | 85 | 102 |
| SeekInCare | 27 | Retrospective + Prospective cohort study | 2400 | cfDNA, fragmentomics, oncogenic virus, 7 serum tumor markers | / | 70 | 95.2 | 10.9 | 71.3 | 103 |
| SPOT-MAS | 5 | Case-control study | 2288 | ctDNA methylation + fragmentation + copy number | / | 72.4 | 97 | / | 73 | 104 |
| SYMPLIFY | >50 | Prospective observational study | 6238 | ctDNA methylation | 5.91 | 66.3 | 98.4 | 75.5 | 85.2 | 105 |
| THEMIS | 7 | Prospective interventional study | 1374 | ctDNA methylation + fragmentation + copy number | 4.8 | / | 99 | 40 | / | 106 |
| thromboSeq | 18 | Case-control study | 2351 | ctRNA | / | 64 | 99 | 99.7 | 68 | 107 |
| THUNDER | 6 | Case-control study | 2395 | ctDNA methylation | / | 69.1 | 98.9 | / | 83.2 | 108 |
| 4-miRNA MCED model | 12 | Case-control study | 8597 | miRNA | / | >90 | 99 | / | / | 109 |
| Urine-MCED metabolomics | 3 | Case-control study | 1703 | Urine metabolomics (GC-MS / UPLC-MS) | / | / | 95 | / | 75 | 110 |
| ctDNA, circulating tumor DNA; cfDNA, cell-free DNA; ctRNA, circulating tumor RNA; MCED, multi-cancer early detection; PPV, positive predictive value; TOO, tissue-of-origin; SERS, surface-enhanced Raman spectroscopy; AI, artificial intelligence; exoRNA, exosomal RNA; GC-MS, gas chromatography–mass spectrometry; UPLC-MS, ultra-performance liquid chromatography–mass spectrometry; PET-CT, positron emission tomography–computed tomography; CNV, copy number variation; CNA, copy number alteration. | | | | | | | | | | |

**References**

[1] Shao X, Jin X, Chen Z, et al. A comprehensive comparison of circulating tumor cells and breast imaging modalities as screening tools for breast cancer in Chinese women. Front Oncol. 2022;12:890248.

[2] Krol I, Schwab FD, Carbone R, et al. Detection of clustered circulating tumour cells in early breast cancer. Br J Cancer. 2021;125(1):23-27.

[3] Tsai WS, You JF, Hung HY, et al. Novel circulating tumor cell assay for detection of colorectal adenomas and cancer. Clin Transl Gastroenterol. 2019;10(10):e00088.

[4] Kang HM, Kim GH, Jeon HK, et al. Circulating tumor cells detected by lab-on-a-disc: Role in early diagnosis of gastric cancer. PLoS One. 2017;12(6):e0180251.

[5] Cheng Y, Luo L, Zhang J, et al. Diagnostic value of different phenotype circulating tumor cells in hepatocellular carcinoma. J Gastrointest Surg. 2019;23(12):2354-2361.

[6] Court CM, Hou S, Winograd P, et al. A novel multimarker assay for the phenotypic profiling of circulating tumor cells in hepatocellular carcinoma. Liver Transpl. 2018;24(7):946-960.

[7] Ilie M, Hofman V, Long-Mira E, et al. "Sentinel" circulating tumor cells allow early diagnosis of lung cancer in patients with chronic obstructive pulmonary disease. PLoS One. 2014;9(10):e111597.

[8] Marquette CH, Boutros J, Benzaquen J, et al. Circulating tumour cells as a potential biomarker for lung cancer screening: a prospective cohort study. Lancet Respir Med. 2020;8(7):709-716.

[9] Ried K, Eng P, Sali A. Screening for circulating tumour cells allows early detection of cancer and monitoring of treatment effectiveness: an observational study. Asian Pac J Cancer Prev. 2017;18(8):2275-2285.

[10] Hofman V, Long E, Ilie M, et al. Morphological analysis of circulating tumour cells in patients undergoing surgery for non-small cell lung carcinoma using the isolation by size of epithelial tumour cell (ISET) method. Cytopathology. 2012;23(1):30-38.

[11] Ankeny JS, Court CM, Hou S, et al. Circulating tumour cells as a biomarker for diagnosis and staging in pancreatic cancer. Br J Cancer. 2016;114(12):1367-1375.

[12] Xu L, Mao X, Grey A, et al. Noninvasive detection of clinically significant prostate cancer using circulating tumor cells. J Urol. 2020;203(1):73-82.

[13] Cao Y, Yu X, Zeng T, et al. Molecular characterization of exosomes for subtype-based diagnosis of breast cancer. J Am Chem Soc. 2022; 144(30):13475-13486.

[14] Moon PG, Lee JE, Cho YE, et al. Identification of developmental endothelial locus-1 on circulating extracellular vesicles as a novel biomarker for early breast cancer detection. Clin Cancer Res. 2016;22(7):1757-1766.

[15] Koo B, Kim YI, Lee M, et al. Enhanced early detection of colorectal cancer via blood biomarker combinations identified through extracellular vesicle isolation and artificial intelligence analysis. J Extracell Vesicles. 2025;14(6):e70088.

[16] Vychytilova-Faltejskova P, Pavlikova M, Pifkova L, et al. Comprehensive profiling of lncRNAs and mRNAs enriched in small extracellular vesicles for early noninvasive detection of colorectal cancer: diagnostic panel assembly and extensive validation. Mol Oncol. 2025;19(11):3445-3462.

[17] Shi Y, Zhuang Y, Zhang J, Chen M, Wu S. Four circulating exosomal miRNAs as novel potential biomarkers for the early diagnosis of human colorectal cancer. Tissue Cell. 2021;70:101499.

[18] Zhang J, Gao Z, Xiao W, et al. A simplified and efficient extracellular vesicle-based proteomics strategy for early diagnosis of colorectal cancer. Chem Sci. 15(44), 18419–18430.

[19] Min L, Bu F, Meng J, et al. Circulating small extracellular vesicle RNA profiling for the detection of T1a stage colorectal cancer and precancerous advanced adenoma. Elife. 2024;12:RP88675.

[20] Tang Y, Zhao Y, Song X, et al. Tumor-derived exosomal miRNA-320d as a biomarker for metastatic colorectal cancer. J Clin Lab Anal. 2019;33(9):e23004.

[21] Pan B, Qin J, Liu X, et al. Identification of serum exosomal hsa-circ-0004771 as a novel diagnostic biomarker of colorectal cancer. Front Genet. 2019;10:1096.

[22] Zhao R, Zhang Y, Zhang X, et al. Exosomal long noncoding RNA HOTTIP as potential novel diagnostic and prognostic biomarker test for gastric cancer. Mol Cancer. 2018;17(1):68.

[23] Yoon JH, Park YG, Nam SW, et al. The diagnostic value of serum gastrokine 1 (GKN1) protein in gastric cancer. Cancer Med. 2019;8(12):5507-5514.

[24] Rui T, Wang K, Xiang A, et al. Serum exosome-derived piRNAs could be promising biomarkers for HCC diagnosis. Int J Nanomedicine. 2023;18:1989-2001.

[25] Zhang Y, Wong CY, Lim CZJ, et al. Multiplexed RNA profiling by regenerative catalysis enables blood-based subtyping of brain tumors. Nat Commun. 2023;14(1):4278.

[26] Yao J, Hua X, Shi J, et al. LncRNA THEMIS2-211, a tumor-originated circulating exosomal biomarker, promotes the growth and metastasis of hepatocellular carcinoma by functioning as a competing endogenous RNA. FASEB J. 2022;36(4):e22238.

[27] Yi K, Wang Y, Rong Y, et al. Transcriptomic signature of 3D hierarchical porous chip enriched exosomes for early detection and progression monitoring of hepatocellular carcinoma. Adv Sci (Weinh). 2024;11(14):e2305204.

[28] Yu Z, Yang Y, Fang W, et al. Dual tumor exosome biomarker co-recognitions based nanoliquid biopsy for the accurate early diagnosis of pancreatic cancer. ACS Nano. 2023;17(12):11384-11395.

[29] Cho HJ, Eun JW, Baek GO, et al. Serum exosomal microRNA, miR-10b-5p, as a potential diagnostic biomarker for early-stage hepatocellular carcinoma. J Clin Med. 2020; 9(1):281.

[30] Kim DH, Park H, Choi YJ, et al. Identification of exosomal microRNA panel as diagnostic and prognostic biomarker for small cell lung cancer. Biomark Res. 2023;11(1):80.

[31] Hua Y, Dai C, He Q, Cai X, Li M. Autoantibody panel on small extracellular vesicles for the early detection of lung cancer. Clin Immunol. 2022;245:109175.

[32] Shin H, ChoH i BH, Shim O, et al. Single test-based diagnosis of multiple cancer types using Exosome-SERS-AI for early stage cancers. Nat Commun. 2023;14(1):1644.

[33] Hajjar M, Aldabbagh G, Albaradei S. Interpretable multi-cancer early detection using SHAP-based machine learning on tumor-educated platelet RNA. Diagnostics (Basel). 2025;15(17):2216.

[34] Yao B, Qu S, Hu R, et al. Delivery of platelet TPM3 mRNA into breast cancer cells via microvesicles enhances metastasis. FEBS Open Bio. 2019;9(12):2159-2169.

[35] Best MG, Sol N, Kooi I, et al. RNA-seq of tumor-educated platelets enables blood-based pan-cancer, multiclass, and molecular pathway cancer diagnostics. Cancer Cell. 2015;28(5):666-676.

[36] Ye B, Li F, Chen M, et al. A panel of platelet-associated circulating long non-coding RNAs as potential biomarkers for colorectal cancer. Genomics. 2022;114(1):31-37.

[37] Xu L, Li X, Li X, et al. RNA profiling of blood platelets noninvasively differentiates colorectal cancer from healthy donors and noncancerous intestinal diseases: a retrospective cohort study. Genome Med. 2022;14(1):26.

[38] Sol N, In 't Veld SGJG, Vancura A, et al. Tumor-educated platelet RNA for the detection and (Pseudo) progression monitoring of glioblastoma. Cell Rep Med. 2020;1(7):100101.

[39] Shao M, Cao J, Wang R, et al. Tumor-educated platelet RNA as a diagnostic biomarker for ground-glass opacity-related lung adenocarcinoma. Transl Lung Cancer Res. 2025;14(8):3090-3106.

[40] Li X, Liu L, Song X, et al. TEP linc-GTF2H2-1, RP3-466P17.2, and lnc-ST8SIA4-12 as novel biomarkers for lung cancer diagnosis and progression prediction. J Cancer Res Clin Oncol. 2021;147(6):1609-1622.

[41] Sheng M, Dong Z, Xie Y. Identification of tumor-educated platelet biomarkers of non-small-cell lung cancer. Onco Targets Ther. 2018;11:8143-8151.

[42] Xing S, Zeng T, Xue N, et al. Development and validation of tumor-educated blood platelets integrin alpha 2b (ITGA2B) RNA for diagnosis and prognosis of non-small-cell lung cancer through RNA-seq. Int J Biol Sci. 2019;15(9):1977-1992.

[43] Dong X, Song X, Ding S, et al. Tumor-educated platelet SNORD55 as a potential biomarker for the early diagnosis of non-small cell lung cancer. Thorac Cancer. 2021;12(5):659-666.

[44] Best MG, Sol N, In 't Veld SGJG, et al. Swarm intelligence-enhanced detection of non-small-cell lung cancer using tumor-educated platelets. Cancer Cell. 2017;32(2):238-252.e9.

[45] Heinhuis KM, In 't Veld SGJG, Dwarshuis G, et al. RNA-sequencing of tumor-educated platelets, a novel biomarker for blood-based sarcoma diagnostics. Cancers (Basel). 2020;12(6):1372.

[46] Eissa S, Matboli M, Essawy NO, et al. Rapid detection of urinary long non-coding RNA urothelial carcinoma associated one using a PCR-free nanoparticle-based assay. Biomarkers. 2015;20(3):212-217.

[47] Chen Y, Xu Y, Li L, et al. Development of a novel circulating miRNA panel for early detection of esophageal cancer. Am J Transl Res. 2025;17(10):8047-8059.

[48] Li M, Zhou Y, Xia T, et al. Circulating microRNAs from the miR-106a-363 cluster on chromosome X as novel diagnostic biomarkers for breast cancer. Breast Cancer Res Treat. 2018;170(2):257-270.

[49] Alkhathami AG, Hadi A, Alfaifi M, et al. Serum-Based lncRNA ANRIL, TUG1, UCA1, and HIT expressions in breast cancer patients. Dis Markers. 2022;2022:9997212.

[50] Ren D, Lin B, Zhang X, et al. Maintenance of cancer stemness by miR-196b-5p contributes to chemoresistance of colorectal cancer cells via activating STAT3 signaling pathway. Oncotarget. 2017;8(30):49807-49823.

[51] Akers JC, Hua W, Li H, et al. A cerebrospinal fluid microRNA signature as biomarker for glioblastoma. Oncotarget. 2017;8(40):68769-68779.

[52] Sayed GI, Solyman M, El Gedawy G, et al. Circulating miRNA's biomarkers for early detection of hepatocellular carcinoma in Egyptian patients based on machine learning algorithms. Sci Rep. 2024;14(1):4989.

[53] Zeng YL, Guo ZY, Su HZ, et al. Diagnostic and prognostic value of lncRNA cancer susceptibility candidate 9 in hepatocellular carcinoma. World J Gastroenterol. 2019;25(48):6902-6915.

[54] Wang JL, Wang X, Yang D, et al. The expression of microRNA-155 in pPlasma and tissue is matched in human laryngeal squamous cell carcinoma. Yonsei Med J. 2016;57(2):298-305.

[55] Zhang R, Jiang Y, Gu J, et al. Diagnostic role of circulating long non-coding RNA LINC00312 in patients with non-small cell lung cancer: a retrospective study. BMC Cancer. 2025;25(1):47.

[56] Sanda MG, Feng Z, Howard DH, et al. Association between combined TMPRSS2:ERG and PCA3 RNA urinary testing and detection of aggressive prostate cancer. JAMA Oncol. 2017;3(8):1085-1093.

[57] Chen X, Li R, Li X, et al. Identification of a four-microRNA panel in serum for screening renal cell carcinoma. Pathol Res Pract. 2021;227:153625.

[58] Wen Q, Wang Y, Li X, et al. Decreased serum exosomal miR-29a expression and its clinical significance in papillary thyroid carcinoma. J Clin Lab Anal. 2021;35(1):e23560.

[59] Urabe F, Matsuzaki J, Takeshita F, et al. Independent verification of circulating miRNA as diagnostic biomarkers for urothelial carcinoma. Cancer Sci. 2022;113(10):3510-3517.

[60] Shi E, Ye J, Zhang R, et al. A combination of circRNAs as a diagnostic tool for discrimination of papillary thyroid cancer. Onco Targets Ther. 2020;13:4365-4372.

[61] Xu C, Jun E, Okugawa Y, et al. A Circulating Panel of circRNA Biomarkers for the Noninvasive and Early Detection of Pancreatic Ductal Adenocarcinoma. Gastroenterology. 2024;166(1):178-190.e16.

[62] Yang Y, Lv W, Shao Y, et al. Circular RNA-Based Molecular Computation Enhances Plasma Biomarker Detection in Biliary Tract Cancer. Angew Chem Int Ed Engl. 2025;64(26):e202505289.

[63] Sun JW, Qiu S, Yang JY, et al. Hsa_circ_0124055 and hsa_circ_0101622 regulate proliferation and apoptosis in thyroid cancer and serve as prognostic and diagnostic indicators. Eur Rev Med Pharmacol Sci. 2020;24(8):4348-4360.

[64] Hu Y, Song Q, Zhao J, et al. Identification of plasma hsa_circ_0008673 expression as a potential biomarker and tumor regulator of breast cancer. J Clin Lab Anal. 2020;34(9):e23393.

[65] Yu Y, Zheng W, Ji C, et al. Tumor-derived circRNAs as circulating biomarkers for breast cancer. Front Pharmacol. 2022;13:811856.

[66] Liu J, Peng X, Liu Y, et al. The diagnostic value of serum exosomal has_circ_0000615 for breast cancer patients. Int J Gen Med. 2021;14:4545-4554.

[67] Lin L, Cai GX, Zhai XM, et al. Plasma-derived extracellular vesicles circular RNAs serve as biomarkers for breast cancer diagnosis. Front Oncol. 2021;11:752651.

[68] He J, Ma S, Kuai S, et al. Circular RNA circAHSA1 serves as a stable serum biomarker for the diagnosis and progression of gastric cancer. Transl Oncol. 2026;63:102620.

[69] Wang J, Mao C, Fang R, et al. Hsa_circ_0002762 may be a new marker for the gastric cancer diagnosis and prognosis. Clin Lab. 2024;70(5):10.

[70] Roy S, Kanda M, Nomura S, et al. Diagnostic efficacy of circular RNAs as noninvasive, liquid biopsy biomarkers for early detection of gastric cancer. Mol Cancer. 2022;21(1):42.

[71] Xu Y, Kong S, Qin X, et al. Comprehensive assessment of plasma circ_0004771 as a novel diagnostic and dynamic monitoring biomarker in gastric cancer. Onco Targets Ther. 2020;13:10063-10074.

[72] Xu P, Xu X, Wu X, et al. CircTMC5 promotes gastric cancer progression and metastasis by targeting miR-361-3p/RABL6. Gastric Cancer. 2022;25(1):64-82.

[73] Ma S, Kong S, Gu X, et al. As a biomarker for gastric cancer, circPTPN22 regulates the progression of gastric cancer through the EMT pathway. Cancer Cell Int. 2021;21(1):44.

[74] Yuan W, Fang R, Mao C, et al. Serum circular RNA hsa_circ_0000702 as a novel biomarker for diagnosis of gastric cancer. J Clin Lab Anal. 2023;37(3):e24842.

[75] Shao Y, Tao X, Lu R, et al. Hsa_circ_0065149 is an indicator for early gastric cancer screening and prognosis prediction. Pathol Oncol Res. 2020;26(3):1475-1482.

[76] Fan L, Cao Q, Liu J, et al. Circular RNA profiling and its potential for esophageal squamous cell cancer diagnosis and prognosis. Mol Cancer. 2019;18(1):16.

[77] He YD, Tao W, He T, et al. A urine extracellular vesicle circRNA classifier for detection of high-grade prostate cancer in patients with prostate-specific antigen 2-10 ng/mL at initial biopsy. Mol Cancer. 2021;20(1):96.

[78] Zhao SY, Wang J, Ouyang SB, et al. Salivary circular RNAs hsa_circ_0001874 and hsa_circ_0001971 as novel biomarkers for the diagnosis of oral squamous cell carcinoma. Cell Physiol Biochem. 2018;47(6):2511-2521.

[79] Hong L, Xu L, Jin L, et al. Exosomal circular RNA hsa_circ_0006220, and hsa_circ_0001666 as biomarkers in the diagnosis of pancreatic cancer. J Clin Lab Anal. 2022;36(6):e24447.

[80] De Paolis E, Perrucci A, Albertini Petroni G, et al. Genomics and epigenomics approaches for the quantification of circulating tumor DNA in liquid biopsy: relevance of a multimodal strategy. Int J Mol Sci. 2025;26(22):10982.

[81] Maansson CT, Nielsen AL, Sorensen BS. Liquid biopsy epigenetics: establishing a molecular profile based on cell-free DNA. Mol Oncol. 2026;20(3):588-610.

[82] Li L, Sun Y. Circulating tumor DNA methylation detection as biomarker and its application in tumor liquid biopsy: advances and challenges. MedComm (2020). 2024;5(11):e766.

[83] Wang J, Huang J, Hu Y, et al. Terminal modifications independent cell-free RNA sequencing enables sensitive early cancer detection and classification. Nat Commun. 2024;15(1):156.

[84] Wang H, Zhan Q, Ning M, et al. Depletion-assisted multiplexed cell-free RNA sequencing reveals distinct human and microbial signatures in plasma versus extracellular vesicles. Clin Transl Med. 2024;14(7):e1760.

[85] Nesselbush MC, Luca BA, Jeon YJ, et al. An ultrasensitive method for detection of cell-free RNA. Nature. 2025;641(8063):759-768.

[86] Kim KH, Yoo BC. Circulating RNA as a Functional Component of Liquid Biopsy in Cancer: Concepts, Classification, and Clinical Applications. Int J Mol Sci. 2026;27(5):2403.

[87] Papier K, Atkins JR, Tong TYN, et al. Identifying proteomic risk factors for cancer using prospective and exome analyses of 1463 circulating proteins and risk of 19 cancers in the UK Biobank. Nat Commun. 2024;15(1):4010.

[88] Wannberg F, Álvez MB, Qvick A, et al. Plasma protein profiling predicts cancer in patients with non-specific symptoms. Nat Commun. 2025;17(1):151.

[89] Moskov M, Hedlund Lindberg J, Lycke M, et al. Deep plasma proteomics identifies and validates an eight-protein biomarker panel that separate benign from malignant tumors in ovarian cancer. Commun Med (Lond). 2025;5(1):230.

[90] Shen H, Liu M, Yang W, et al. Exosomal proteins: new targets for early diagnosis and treatment of cancer. Front Immunol. 2025;16:1613494.

[91] Kamli H, Khan NU. Emerging role of ctDNA fragmentomics and epigenetic signatures in the early detection, minimal residual disease assessment, and precision monitoring of renal cell carcinoma. J Cell Mol Med. 2026;30(3):e71019.

[92] Cohen JD, Li L, Wang Y, et al. Detection and localization of surgically resectable cancers with a multi-analyte blood test. Science. 2018;359(6378):926-930.

[93] Liu MC, Oxnard GR, Klein EA, et al. Sensitive and specific multi-cancer detection and localization using methylation signatures in cell-free DNA. Ann Oncol. 2020;31(6):745-759.

[94] Cristiano S, Leal A, Phallen J, et al. Genome-wide cell-free DNA fragmentation in patients with cancer. Nature. 2019;570(7761):385-389.

[95] Lennon AM, Buchanan AH, Kinde I, et al. Feasibility of blood testing combined with PET-CT to screen for cancer and guide intervention. Science. 2020;369(6499):eabb9601.

[96] Wang F, Wang C, Chen S, et al. Identification of blood-derived exosomal tumor RNA signatures as noninvasive diagnostic biomarkers for multi-cancer: a multi-phase, multi-center study. Mol Cancer. 2025;24(1):60.

[97] Yang X, He D, Xiong Z, et al. Early detection and localization of major gastrointestinal cancers by combining DNA methylation and fragmentomic signatures generated from a single cfDNA targeted sequencing assay. JCO 2023;41:330.

[98] Nguyen LHD, Nguyen THH, Le VH, et al. Prospective validation study: a non-invasive circulating tumor DNA-based assay for simultaneous early detection of multiple cancers in asymptomatic adults. BMC Med. 2025;23(1):90.

[99] Bao H, Yang S, Chen X, et al. Early detection of multiple cancer types using multidimensional cell-free DNA fragmentomics. Nat Med. 2025;31(8):2737-2745.

[100] Luan Y, Zhong G, Li S, et al. A panel of seven protein tumour markers for effective and affordable multi-cancer early detection by artificial intelligence: a large-scale and multicentre case-control study. EClinicalMedicine. 2023;61:102041.

[101] Chen X, Gole J, Gore A, et al. Non-invasive early detection of cancer four years before conventional diagnosis using a blood test. Nat Commun. 2020;11(1):3475.

[102] Schrag D, Beer TM, McDonnell CH 3rd, et al. Blood-based tests for multicancer early detection (PATHFINDER): a prospective cohort study. Lancet. 2023;402(10409):1251-1260.

[103] Li S, Geng S, Chen Y, et al. Clinical validation of a noninvasive multi-omics method for multicancer early detection in retrospective and prospective cohorts. J Mol Diagn. 2025;27(7):657-670.

[104] Nguyen VTC, Nguyen TH, Doan NNT, et al. Multimodal analysis of methylomics and fragmentomics in plasma cell-free DNA for multi-cancer early detection and localization. Elife. 2023;12:RP89083.

[105] Nicholson BD, Oke J, Virdee PS, et al. Multi-cancer early detection test in symptomatic patients referred for cancer investigation in England and Wales (SYMPLIFY): a large-scale, observational cohort study. Lancet Oncol. 2023;24(7):733-743.

[106] Li Y, Song X, Shen N, et al. Real-world performance of a multi-cancer early detection test based on integrative analysis of cell-free DNA whole methylome sequencing. J Clin Oncol 2023;41:10521.

[107] In 't Veld SGJG, Arkani M, Post E, et al. Detection and localization of early- and late-stage cancers using platelet RNA. Cancer Cell. 2022;40(9):999-1009.e6.

[108] Gao Q, Lin YP, Li BS, et al. Unintrusive multi-cancer detection by circulating cell-free DNA methylation sequencing (THUNDER): development and independent validation studies. Ann Oncol. 2023;34(5):486-495.

[109] Zhang J, Rui H, Hu H. Noninvasive multi-cancer detection using blood-based cell-free microRNAs. Sci Rep. 2024;14(1):22136.

[110] Xu X, Zeng C, Qing B, et al. Development of a urine-based metabolomics approach for multi-cancer screening and tumor origin prediction. Front Immunol. 2024;15:1449103.
